# Cell-free DNA maps COVID-19 tissue injury and risk of death and can cause tissue injury

**DOI:** 10.1172/jci.insight.147610

**Published:** 2021-04-08

**Authors:** Temesgen E. Andargie, Naoko Tsuji, Fayaz Seifuddin, Moon Kyoo Jang, Peter S.T. Yuen, Hyesik Kong, Ilker Tunc, Komudi Singh, Ananth Charya, Kenneth Wilkins, Steven Nathan, Andrea Cox, Mehdi Pirooznia, Robert A. Star, Sean Agbor-Enoh

**Affiliations:** 1 Genomic Research Alliance for Transplantation (GRAfT) and Laboratory of Applied Precision Omics, National Heart, Lung, and Blood Institute (NHLBI), NIH, Bethesda, Maryland, USA.; 2 Department of Biology, Howard University, Washington DC, USA.; 3 Renal Diagnostics and Therapeutics Unit, National Institute of Diabetes and Digestive and Kidney Diseases (NIDDK), NIH, Bethesda, Maryland, USA.; 4 Bioinformatics and Computation Core, NHLBI, Maryland, USA.; 5 Office of the Director, NIDDK, NIH, Bethesda, Maryland, USA.; 6 Advanced Lung Disease and Transplant Program, Inova Fairfax Hospital, Fairfax, Virginia, USA.; 7 Department of Medicine, Johns Hopkins University School of Medicine, Baltimore, Maryland, USA.

**Keywords:** COVID-19, Inflammation, Bioinformatics, Molecular genetics

## Abstract

**INTRODUCTION:**

The clinical course of coronavirus 2019 (COVID-19) is heterogeneous, ranging from mild to severe multiorgan failure and death. In this study, we analyzed cell-free DNA (cfDNA) as a biomarker of injury to define the sources of tissue injury that contribute to such different trajectories.

**METHODS:**

We conducted a multicenter prospective cohort study to enroll patients with COVID-19 and collect plasma samples. Plasma cfDNA was subject to bisulfite sequencing. A library of tissue-specific DNA methylation signatures was used to analyze sequence reads to quantitate cfDNA from different tissue types. We then determined the correlation of tissue-specific cfDNA measures to COVID-19 outcomes. Similar analyses were performed for healthy controls and a comparator group of patients with respiratory syncytial virus and influenza.

**RESULTS:**

We found markedly elevated levels and divergent tissue sources of cfDNA in COVID-19 patients compared with patients who had influenza and/or respiratory syncytial virus and with healthy controls. The major sources of cfDNA in COVID-19 were hematopoietic cells, vascular endothelium, hepatocytes, adipocytes, kidney, heart, and lung. cfDNA levels positively correlated with COVID-19 disease severity, C-reactive protein, and D-dimer. cfDNA profile at admission identified patients who subsequently required intensive care or died during hospitalization. Furthermore, the increased cfDNA in COVID-19 patients generated excessive mitochondrial ROS (mtROS) in renal tubular cells in a concentration-dependent manner. This mtROS production was inhibited by a TLR9-specific antagonist.

**CONCLUSION:**

cfDNA maps tissue injury that predicts COVID-19 outcomes and may mechanistically propagate COVID-19–induced tissue injury.

**FUNDING:**

Intramural Targeted Anti–COVID-19 grant, NIH.

## Graphical abstract

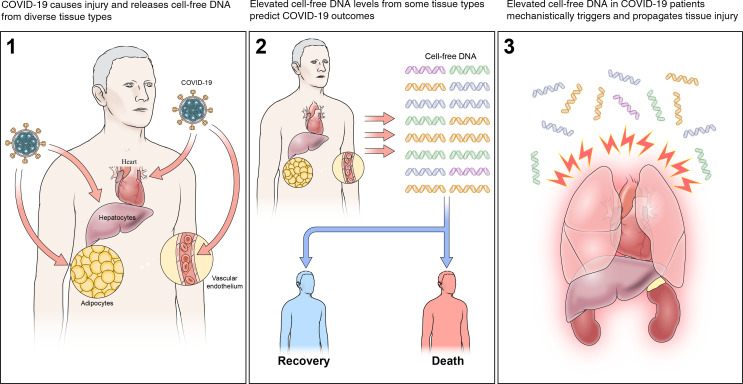

## Introduction

Coronavirus disease 2019 (COVID-19) is a global pandemic caused by the novel SARS coronavirus 2 (SARS-CoV-2) that has resulted in 71.3 million confirmed cases and over 1.6 million fatalities as of December 12, 2020 (1). Symptoms are often mild or absent at disease onset. The subsequent clinical trajectories vary significantly, imposing significant challenges to health care resources; most patients infected with SARS-CoV-2 develop mild illnesses, recovering quickly and without the need for hospitalization, whereas others develop severe disease requiring hospitalization. In the latter group, some patients require general medicine admission and others have progressive disease that necessitates intensive care unit (ICU) care (2). Although the lung is the predominant locus of serious SARS-CoV-2 infections, multiple organs and tissues can become involved (3, 4), including widespread damage to the vasculature, heart, kidney, and other organs (5, 6). The sources of tissue injury that contribute to these different clinical trajectories remain poorly defined.

In several diseases, cell-free DNA (cfDNA), including nuclear (ncfDNA) and mitochondrial (mtcfDNA), is released into the circulation after apoptosis, necrosis, and active secretion from cells (7). In a healthy individual, cfDNA is present in small amounts (8), has a short half-life (9), and is predominantly derived from circulating hematopoietic cells. The composition and quantity of cfDNA dramatically changes during pathological conditions. Indeed, several studies reported elevated cfDNA concentration as a potential noninvasive biomarker in many diseases (10) and transplant rejection (11–13). Moreover, cfDNA biomarkers have the potential to detect disease before clinical manifestations or histopathological changes develop, as in the case of transplant rejection. Furthermore, circulating cfDNA concentration predicts long-term outcomes in lung transplantation and other diseases. Since cfDNA maintains the methylation signatures of its tissues of origin (14, 15), cfDNA methylomic analysis may enable mapping of the sources of tissue injury that contribute to and predict different COVID-19 clinical trajectories.

Beyond direct infection of cells by SARS-CoV2, subsequent triggers of tissue or organ injury remain poorly defined in COVID-19. Although the SARS-CoV-2 receptor, angiotensin-converting enzyme 2 (ACE2), is expressed in the lung, heart, kidney, intestine, and vascular endothelium (5, 16–20), injury is often detected in these tissues without detectable viral particles (21). Additionally, injury has also been detected in cells of types that do not express the ACE2 receptor, implicating nonviral triggers of tissue injury (22). Recent studies reported that dysregulation of immune response and uncontrolled inflammation are associated with tissue injury in COVID-19 (23, 24), such as increased proinflammatory monocyte-derived macrophages (25); impaired type I IFN response; and elevated TNF-α, IL-6, and IL-8 production (26, 27). However, little is known about the driving factors that lead to excessive inflammation in COVID-19 patients.

In addition to being a biomarker for tissue injury, cfDNA may act as a danger-associated molecular pattern (DAMP) and aggravate inflammation (28). mtcfDNA, containing short nucleosome-free and high CpG islands (29), binds to TLR9 and triggers a proinflammatory response. We have also previously shown that mtcfDNA contributes to cytokine production, apoptosis, and tubular mitochondrial injury via TLR9 in sepsis-induced acute kidney injury (AKI, ref. 30). Moreover, circulatory cfDNA has also been involved in coagulation activation and thrombus formation in sepsis patients (31). Recent studies have also reported elevated levels of cfDNA in COVID-19 patients that are strongly correlated with acute-phase reactants, neutrophil count, and disease progression (32). cfDNA is also a critical component of neutrophil-derived extracellular traps (NETs), which are elevated in COVID-19 patients (32–34).

In this study, we leveraged cfDNA to map the sources of tissue injury that correlate with COVID-19 clinical trajectories and outcomes and assessed whether cfDNA released with COVID-19 can act as a DAMP.

## Results

### Demographics and clinical characteristics of patients with COVID-19.

**Figure 1:**
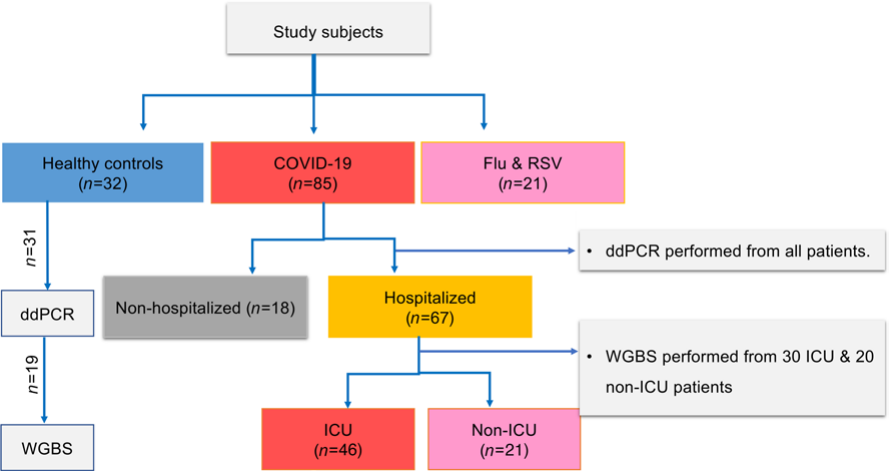
Flow diagram showing patient selection and experiments. Out of 85 enrolled patients with COVID-19 (67 hospitalized and 18 nonhospitalized), 6 hospitalized patients were excluded from analysis for absence of admission samples. Total ncfDNA and mtcfDNA were quantified from all study subjects via digital droplet PCR (ddPCR). A subset of hospitalized patients (*n =* 50: 30 ICU and 20 non-ICU) were subjected to whole genome bisulfite sequencing (WGBS). Among the 32 healthy controls enrolled, ddPCR and WGBS were performed in 31 and 19 healthy controls, respectively. All the patients with influenza or RSV were subjected to ddPCR and WGBS.

We recruited 85 patients with COVID-19 (Table 1 and Figure 1) and grouped them by their maximum disease severity during their COVID-19 illness using the WHO scale, an 8-point ordinal scale measurement for clinical improvement, which is based on health care resource utilization and death (35). The cohort included 18 nonhospitalized (WHO scale 1, 2) and 67 hospitalized patients, including 21 hospitalized patients not requiring ICU care (WHO scale 3, 4) and 46 patients requiring ICU care (WHO scale 5–8) during their COVID-19 illness. The median age (IQR) was 48.5 (ages 37–56) years and 64.7% were male. Most of the hospitalized patients (*n =* 52, 77.6%) had at least 1 underlying comorbidity: hypertension (*n =* 27, 41%), diabetes mellitus (*n =* 28, 42.4%), and obesity (*n =* 25, 37.9%) were the most common. Sex and race showed a significant difference between ICU and non-ICU patients. Eighteen hospitalized patients (27.4%) died. In addition, 21 patients infected with influenza and/or respiratory syncytial virus (RSV) were included as comparator group. This group was matched for disease severity using WHO scale for disease progression (Supplemental Table 1; supplemental material available online with this article; https://doi.org/10.1172/jci.insight.147610DS1). We also included 32 healthy controls.

### Elevated cfDNA levels in COVID-19 patients.

**Figure 2:**
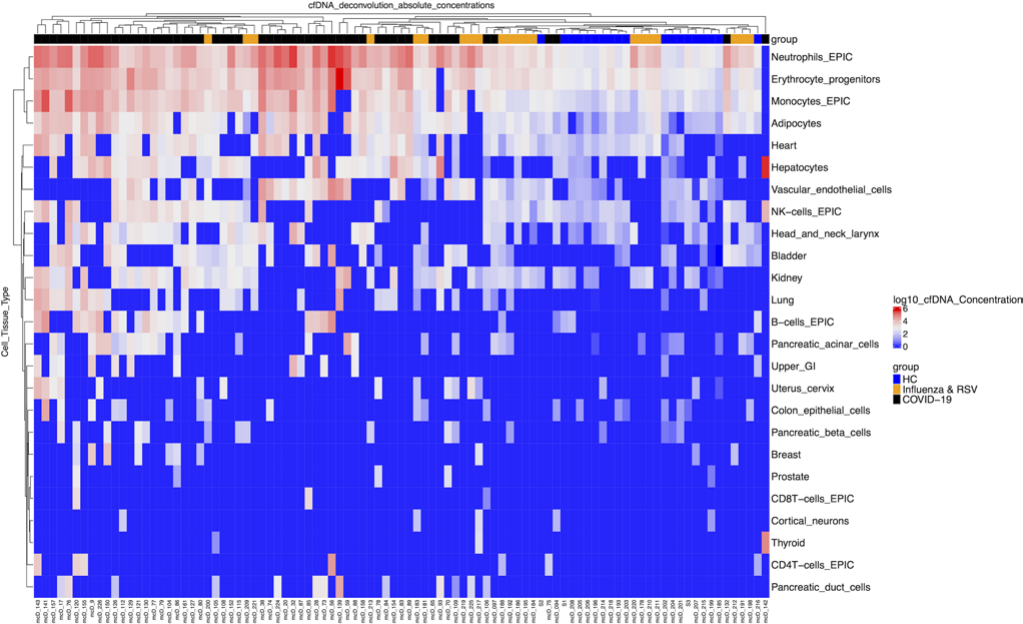
Absolute concentrations of cfDNA from different tissue types in patients. Heatmap plot showing unsupervised clustering of cfDNA concentrations from different tissue sources (copies/mL in log scale), each column representing individual patients, which include COVID-19 patients (*n =* 55, black), healthy controls (*n =* 19, blue), and a comparator group of patients with influenza or respiratory syncytial virus (*n =* 21, orange). cfDNA from individual tissue types were quantified by bisulfite sequencing, analyzing tissue-specific DNA methylation signatures. cfDNA concentration is represented in copies/mL of plasma and reported in log scale from lowest (blue) to highest (red) values.

**Figure 3:**
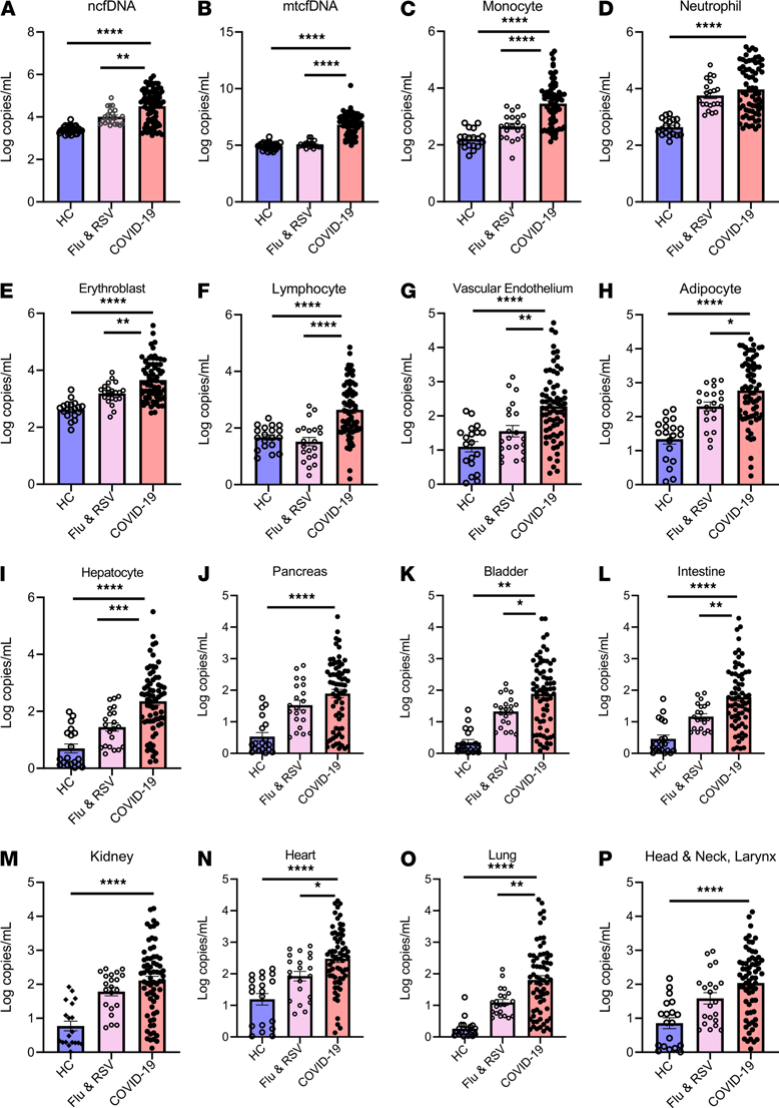
Circulating cfDNA concentration is markedly elevated in patients with COVID-19. Concentration of plasma nuclear cell-free DNA (ncfDNA) (**A**) and mitochondrial cell-free cfDNA (mtcfDNA) (**B**) for healthy controls (HC, blue, *n =* 31), patients with influenza or RSV (pink, *n =* 21), and COVID-19 patients (light red, *n =* 79). (**C**–**P**) Identification of cell/tissue-contributor of cfDNA by using deconvolution of tissue-specific DNA methylation signatures on cfDNA: (**C**) monocyte-, (**D**) neutrophil-, (**E**) erythroblast-, (**F**) lymphocyte-, (**G**) vascular endothelial cell-, (**H**) adipocyte-, (**I**) hepatocyte-, (**J**) pancreas-, (**K**) bladder-, (**L**) colon enterocyte-, (**M**) kidney-, (**N**) heart-, (**O**) lung-, and (**P**) head and neck larynx-derived cfDNA in HCs, influenza and RSV patients, and COVID-19 patients. Bar graphs expressed as mean ± SEM. Statistical significance levels for each pairwise comparison were determined using adjusted *P* values computed based on Hommel’s procedure. Adjusted *P* values are shown. *P* values less than 0.05 were considered statistically significant; *P < 0.05, **P < 0.01, ***P < 0.001, and ****P < 0.0001.

We quantified total (bulk) and mtcfDNA by digital droplet PCR and next performed bisulfite sequencing and analyzed cfDNA sequence reads using a deconvolution algorithm and a library of tissue-specific DNA methylation signatures to investigate the sources of cfDNA in patients with COVID-19, patients with influenza and/or RSV, and healthy controls (Figure 2). COVID-19 infection markedly elevated (20-fold) total (bulk) ncfDNA (47,500 copies/mL) compared with healthy controls (2,320 copies/mL, *P <* 0.0001, Figure 3A). Similarly, COVID-19 patients had 250-fold higher mtcfDNA compared with healthy controls (Figure 3B). Overall, hematopoietic cells were the major contributors of cfDNA in the COVID-19 group (~84.5%) and healthy controls (~90.5%), with neutrophils, erythrocyte progenitor cells, and monocytes being the largest contributors. The tissue source of cfDNA was different in COVID-19 patients compared with healthy controls. We found 35- to 390-fold higher cfDNA levels derived from hematopoietic cells in COVID-19 patients compared with healthy controls, including monocytes (14,000 vs. 208 copies/mL), neutrophils (43,250 vs. 531 copies/mL), erythroblasts (19,160 vs. 535 copies/mL), and lymphocytes (3,680 vs. 65 copies/mL); *P <* 0.0001 for all comparisons (Figure 3, C–F). Similarly, the levels of nonhematopoietic tissue–derived cfDNA were approximately 10- to 1000- fold higher in COVID-19 patients compared with healthy controls. Of these, cfDNA derived from the vascular endothelium (2,480 vs. 30 copies/mL), adipocytes (2795 vs. 44.2 copies/mL), hepatocytes (6,860 vs. 15.3 copies/mL), pancreas (762 vs. 7.9 copies/mL), bladder (915 vs. 2.8 copies/mL), intestine (745 vs. 6.1 copies/mL), kidney (1,152 vs. 15.1 copies/mL), heart (1,824 vs. 42.5 copies/mL), lung (1098 vs. 1.25 copies/mL), and head and neck larynx (715 vs. 21.7 copies/mL) were higher in COVID-19 patients compared with healthy controls; *P <* 0.0001 for all comparisons (Figure 3, G–P).

We next asked whether the cfDNA (bulk or tissue source) in COVID-19 patients differed from other respiratory viral infections (influenza and RSV). The median levels of ncfDNA and mtcfDNA were 4.7-fold and 67-fold higher in COVID-19 patients compared with patients with influenza and RSV, respectively; *P <* 0.0001. (Figure 3, A and B). To control for disease severity, we compared WHO scale–matched patients; hospitalized COVID-19 patients had 3-fold higher ncfDNA and 410-fold higher mtcfDNA compared with the influenza and RSV group, respectively; *P <* 0.0001 for both comparisons (Supplemental Figure 1, A and B). Similarly, tissue-specific cfDNA derived from monocytes, erythroblasts, lymphocytes, vascular endothelium, adipocytes, hepatocytes, pancreas, bladder, and lung was 2- to 11-fold higher in hospitalized COVID-19 patients compared with hospitalized patients with influenza and RSV; *P <* 0.05 for all comparisons (Supplemental Figure 1, C–P).

### cfDNA profile predicts COVID-19 patient outcomes.

**Figure 4:**
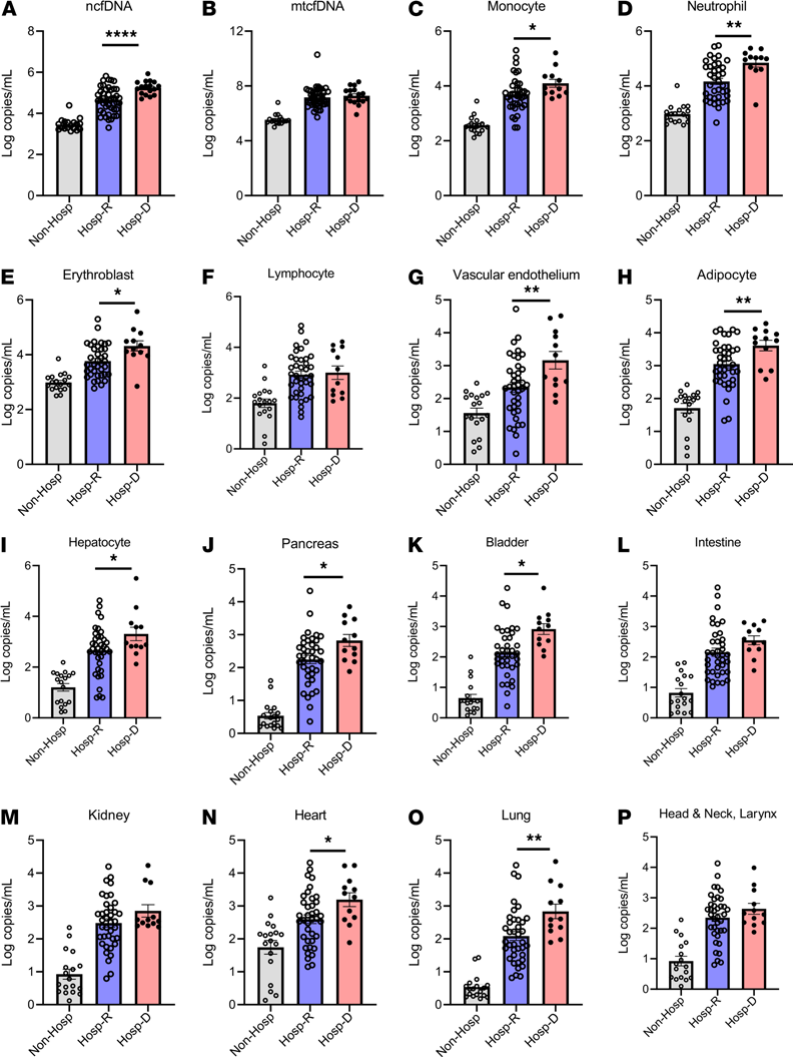
Plasma cfDNA levels by COVID-19 outcome. Patients were stratified by COVID-19 outcomes as nonhospitalized, representing patients with mild disease who did not require hospitalization (Non-Hosp), gray (*n =* 18); hospitalized patients who recovered (Hosp-R), light blue (*n =* 48); or hospitalized who died (Hosp-D), light red (*n =* 18). (**A**) Nuclear cfDNA (ncfDNA), (**B**) mitochondrial cfDNA (mtcfDNA), and cfDNA from different tissue types (**C**–**P**) are shown for the 3 groups of COVID-19 patients. Only cfDNA measurements early in the COVID-19 illness were considered (the first sample per patient, typically days 0–2 of admission for hospitalized patients or close to testing for patients with mild COVID-19). The following tissue types are shown: (**C**) monocytes, (**D**) neutrophils, (**E**) erythroblasts, (**F**) lymphocytes, (**G**) vascular endothelial cells, (**H**) adipocytes, (**I**) hepatocytes, (**J**) pancreas, (**K**) bladder, (**L**) colon enterocytes, (**M**) kidney, (**N**) heart, (**O**) lung, and (**P**) head and neck larynx. Bar graphs expressed as mean ± SEM. Statistical significance levels for each pairwise comparison were determined using adjusted *P* values computed based on Hommel’s procedure. Adjusted *P* values are shown. *P* values less than 0.05 were considered statistically significant; *P < 0.05, **P < 0.01, and ****P < 0.0001.

Within COVID-19 groups, we observed different amounts of total cfDNA, mtcfDNA, and cfDNA from different tissue types (Figure 2); cfDNA profiles correlated with concurrent measure of disease severity as assessed by WHO scale. Next, we sought to determine whether cfDNA measurements on admission can predict subsequent patient outcomes, including death. Only the first sample per patient was considered and we only included patients who had a first sample drawn within 0–2 days of admission, arbitrarily selected to represent early admission. In total, 61 patients met this inclusion criteria, including 17 patients who died and 44 patients who survived. We found plasma ncfDNA levels within the first 2 days of hospital admission were 4.5-fold higher in patients who eventually died of COVID-19 compared with patients who recovered (196,790 vs. 43,470 copies/mL, *P <* 0.0001; Figure 4A). The mtcfDNA level did not significantly differ in patients who later died compared with patients who recovered (*P =* 0.5291; Figure 4B). We further compared tissue injury profiles in the 2 COVID-19 patient groups: those who died from the disease versus those who survived. Elevated mean concentrations of cfDNA derived from monocytes (8,418 vs. 4,245 copies/mL), neutrophils (106,400 vs. 12,920 copies/mL), erythroblasts (21,660 vs. 4,606 copies/mL), vascular endothelium (7,141 vs. 2,146 copies/mL), adipocytes (6,187 vs. 1,147 vs. copies/mL), hepatocytes (29,300 vs. 3,012 copies/mL), pancreas (5,732 vs. 857 copies/mL), bladder (1,434 vs. 907 copies/mL), heart (2,140 vs. 339 copies/mL), and lung (2,925 vs. 1,040 copies/mL) were observed in patients who later died compared with survivors; *P <* 0.05 for all comparisons. Although not statistically significant, there was a higher level of cfDNA derived from the intestine, kidney, and head and neck larynx (Figure 4, C–P).

We next performed a multivariate analysis examining the association between ncfDNA and outcome (deceased versus recovered), correcting for clinical and demographic covariates. Variables included for comparison were sex, age, obesity, and presence of cancer. These 4 covariates met the threshold for statistical significance in the univariate analysis (Table 1) to be included in subsequent multivariate analysis. Additionally, these variables were deemed clinically relevant as well; older age, obesity, and male sex were all associated with more severe clinical trajectory. After adjustment for these covariates, ncfDNA remained significantly associated with death (OR = 13.3, 95% CI 2.2–82.1, *P =* 0.0053; Supplemental Table 2).

**Figure 5:**
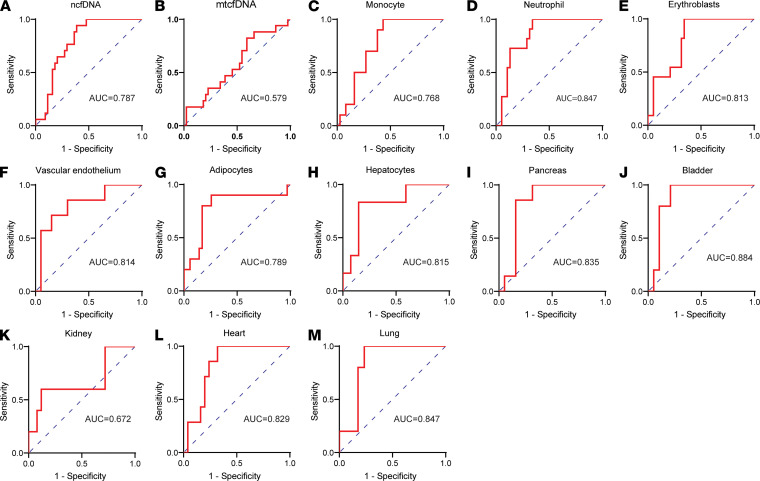
cfDNA profile distinguishes patients with COVID-19 by outcomes. Receiver operating characteristic (ROC) curve analysis of cfDNA measures early at admission as predictor and COVID-19 outcomes categorized as hospitalized recovered and deceased: (**A**) ncfDNA, (**B**) mtcfDNA, (**C**) monocyte-derived cfDNA, (**D**) neutrophil-derived cfDNA, (**E**) erythroblast-derived cfDNA, (**F**) vascular endothelium–derived cfDNA, (**G**) adipocyte-derived cfDNA, (**H**) hepatocyte-derived cfDNA, (**I**) pancreas-derived cfDNA, (**J**) bladder-derived cfDNA, (**K**) kidney-derived cfDNA, (**L**) heart-derived cfDNA, and (**M**) lung-derived cfDNA.

We then assessed the predictive capacity of cfDNA profiles at patient admission using receiver operating characteristic curve (ROC) analysis. The total ncfDNA distinguished patients who eventually died versus those who survived with an AUC of 0.787 (95% CI = 0.676–0.899, *P <* 0.0001; Figure 5A), whereas mtcfDNA showed nonsignificant performance capacity (AUC = 0.579; 95% CI = 0.420–0.738, *P =* 0.3426; Figure 5B). We observed high performance for the following tissue-specific cfDNA as well: monocytes (AUC = 0.768; 95% CI = 0.634–0.901), neutrophils (AUC = 0.847; 95% CI = 0.738–0.956), erythroblasts (AUC = 0.813, 95% CI = 0.691–0.936), vascular endothelium (AUC = 0.814, 95% CI = 0.626–1.000), adipocytes (AUC = 0.789, 95% CI = 0.605–0.971), hepatocytes (AUC = 0.815, 95% CI = 0.632–0.997), pancreas (AUC = 0.835, 95% CI = 0.741–1.000), bladder (AUC = 0.884, 95% CI = 0.748-1.000), heart (AUC = 0.829, 95% CI = 0.688–0.967), and lung (AUC = 0.847, 95% CI = 0.682–1.000) (Figure 5, C–M).

**Figure 6:**
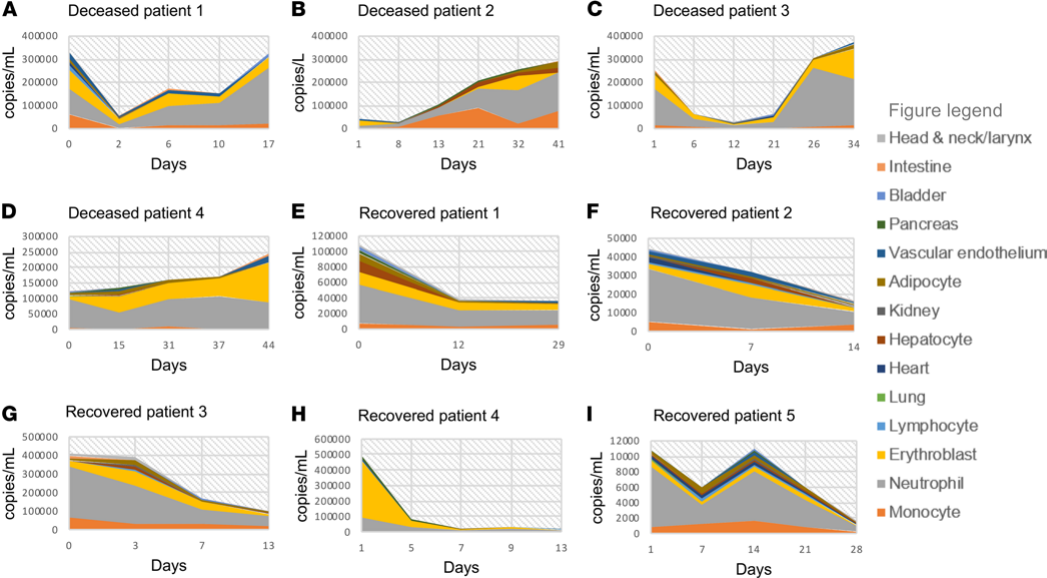
Kinetics of cfDNA profiles for patients by COVID-19 outcome. Changes in tissue-specific cfDNA measures over time for prototype COVID-19 patients who required intensive care and recovered (Recovered) or died (Deceased); an increasing cfDNA trend in Deceased (**A**–**D**) and a decreasing cfDNA trend in Recovered (**E**–**I**). cfDNA concentration in copies/mL is represented in the *y* axis and days from hospital admission represented in the *x* axis (day 0 representing the day of hospital admission). cfDNA from individual tissue type is shown and a figure key is represented.

We further determined the longitudinal changes of cfDNA profile during the course of severe COVID-19, focusing on COVID-19 ICU patients who died compared with ICU patients who survived. The levels of ncfDNA and the tissue-specific cfDNA levels were higher and sustained in the critically ill COVID-19 patients who eventually died compared with those who recovered (Figure 6).

### cfDNA profile differentiates COVID-19 patients by clinical trajectory.

**Figure 7:**
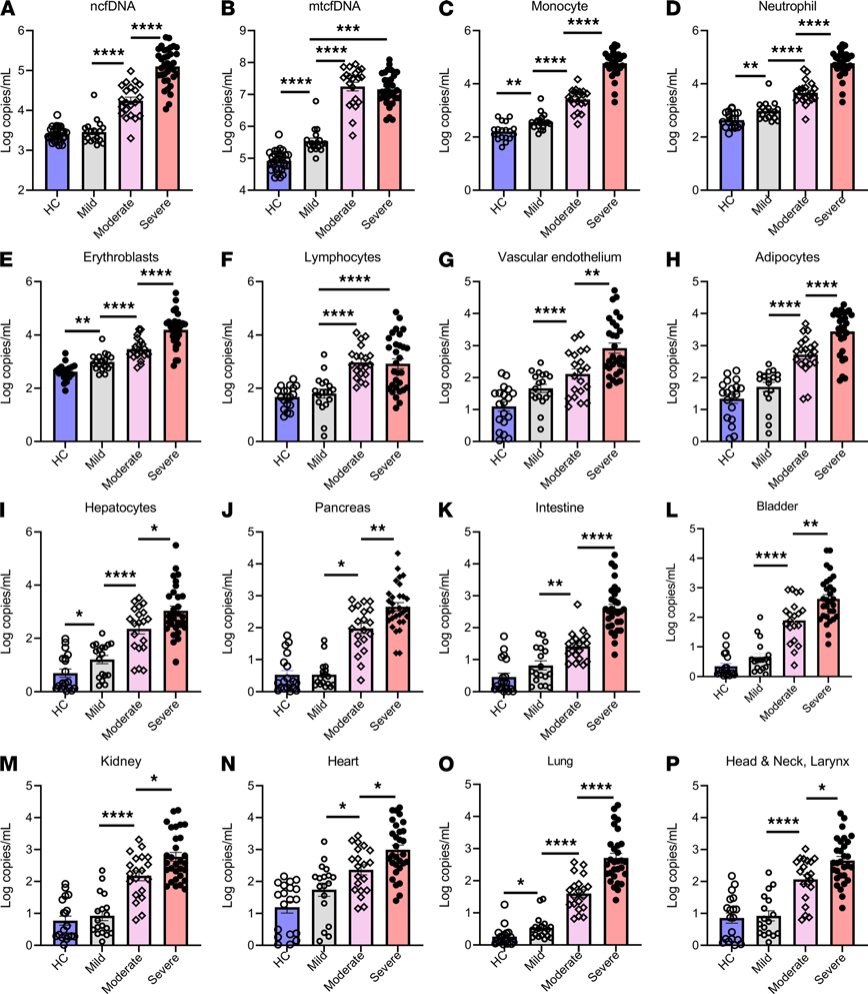
Plasma cfDNA levels for COVID-19 patients by severity. (**A** and **B**) Plasma cfDNA measures compared for healthy controls (HC) shown in light blue (*n =* 31) and COVID-19 patients. COVID-19 patients were categorized by maximum disease severity during their illness using the WHO scale; 1–2 represents patients with mild disease not requiring hospitalization (mild) shown in gray (*n =* 18), 3–4 represents hospitalized patients not requiring intensive care (moderate) shown in light pink (*n =* 20), and 5–8 represents hospitalized patients requiring intensive care unit care (severe) shown in light red (*n =* 30). Only cfDNA measurements early in the COVID-19 course were considered (the first sample per patient, typically days 0–2 of admission for hospitalized patients or close to testing for patients with mild COVID-19). **C**–**K** represent cfDNA from different tissue types grouped by patient categories: (**C**) monocytes, (**D**) neutrophils, (**E**) erythroblasts, (**F**) lymphocytes, (**G**) vascular endothelial cells, (**H**) adipocytes, (**I**) hepatocytes, (**J**) pancreas, (**K**) bladder, (**L**) colon enterocytes, (**M**) kidney, (**N**) heart, (**O**) lung, and (**P**) head and neck larynx. Bar graphs expressed as mean ± SEM. Statistical significance levels for each pairwise comparison were determined using adjusted *P* values computed based on Hommel’s procedure. Adjusted *P* values are shown. *P* values less than 0.05 were considered statistically significant; *P < 0.05, **P < 0.01, ***P < 0.001, ****P < 0.0001.

To assess whether cfDNA level distinguished patients based on their clinical trajectory, we categorized patients based on their maximum WHO scale during their illness as nonhospitalized mild cases (WHO scale 1, 2), moderate/hospitalized non-ICU (WHO scale 3, 4), and severe/hospitalized ICU (WHO scale 5–8). Analyzing the first sample for each patient within days 0 to 2 of admission, patients with severe COVID-19 had elevated ncfDNA on admission (152,600 copies/mL) compared with moderate disease (15,790 copies/mL) and mild cases (2,750 copies/mL); *P <* 0.0001 for both comparisons (Figure 7A). Patients with severe and moderate COVID-19 had significantly higher mtcfDNA levels than nonhospitalized patients with mild COVID-19, *P <* 0.001. However, mtcfDNA levels were not significantly different between patients with moderate and severe COVID-19 (Figure 7B). More importantly, the cfDNA (copies/mL) tissues of origin differed: patients with severe COVID-19 had an increased level of ncfDNA (copies/mL) derived from monocytes (8,114 vs. 3,429), neutrophils (74,300 vs. 4,142), erythroblasts (17,960 vs. 2,521), vascular endothelium (5325 vs. 375), adipocytes (5,600 vs. 1,025), hepatocytes (15,040 vs. 746), pancreas (1,925 vs. 215), intestine (1,657 vs. 35), bladder (1,583 vs. 216), kidney (2,345 vs. 374), heart (3,609 vs. 600), lung (2,425 vs. 94), and head and neck (1,439 vs. 252) compared with hospitalized non-ICU COVID-19 patients; *P <* 0.05 for all. Patients with mild disease showed significantly lower tissue-specific cfDNA, including monocyte (504 copies/mL), neutrophil (1462 copies/mL), erythroblast (1335 copies/mL), lymphocyte (203 copies/mL), vascular endothelium (74 copies/mL), adipocyte (88 copies/mL), hepatocyte (32 copies/mL), pancreas (3 copies/mL), bladder (9 copies/mL), intestine (12 copies/mL), kidney (28 copies/mL), lung (2 copies/mL), and head and neck (22 copies/mL) compared with both severe and moderate cases, *P <* 0.05 for all. Additionally, patients with mild COVID-19 had higher levels of mtcfDNA and cfDNA derived from monocytes, neutrophils, erythroblasts, hepatocytes, bladder, heart, and lung than healthy controls as observed in patients with severe disease, *P <* 0.05; Figure 7, C–P.

**Figure 8:**
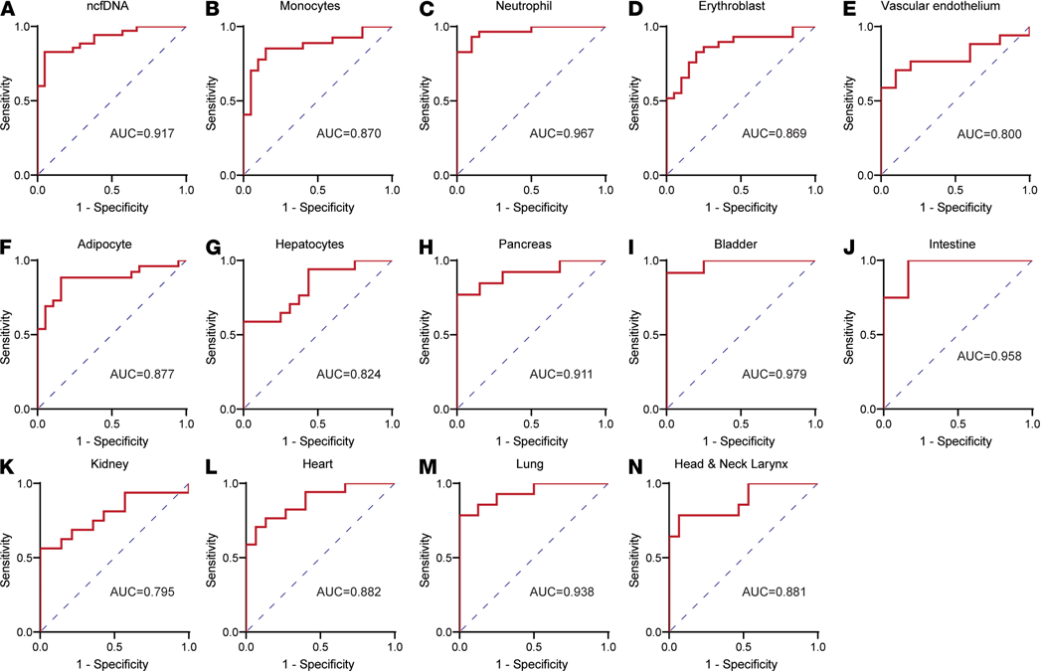
cfDNA profile distinguishes patients by COVID-19 severity. Receiver operating characteristic (ROC) curve analysis of cfDNA measures early at admission as predictor and maximum COVID-19 severity during illness as outcome variable. ROC analysis showing performance of cfDNA profiles to distinguish hospitalized patients who subsequently required ICU versus hospitalized patients who did not: (**A**) ncfDNA-, (**B**) monocyte-, (**C**) neutrophil-, (**D**) erythroblast-, (**E**) vascular endothelium– (**F**) adipocyte-, (**G**) hepatocyte-, (**H**) pancreas, (**I**) bladder-, (**J**) intestine-, (**K**) kidney-, (**L**) heart-, (**M**) lung-, and (**N**) head and neck larynx-derived cfDNA.

We then tested the performance of early admission cfDNA profiles to identify hospitalized patients who required ICU versus patients who do not. The ROC analysis demonstrated that plasma ncfDNA showed high discriminative performance between ICU patients and hospitalized non-ICU patients (AUC = 0.917, 95% CI = 0.847–0.987, *P <* 0.0001; Figure 8A), whereas mtcfDNA did not distinguish ICU versus non-ICU COVID-19 patients. The tissue-specific cfDNA profile also showed a high discriminative performance between ICU and non-ICU patients in cfDNA derived from monocytes (AUC = 0.870; 95% CI = 0.765–0.976), neutrophils (AUC = 0.967; 95% CI = 0.924–1.000), erythroblasts (AUC = 0.869, 95% CI = 0.761–0.967), vascular endothelium (AUC = 0.800, 95% CI = 0.631–0.696), hepatocytes (AUC = 0.824, 95% CI = 0.682–0.965), adipocytes (AUC = 0.877, 95% CI = 0.771–0.982), pancreas (AUC = 0.911, 95% CI = 0.793–1.000), bladder (AUC = 0.979, 95% CI = 0.931–1.000), intestine (AUC = 0.958, 95% CI = 0.866–1.000), kidney (AUC = 0.795, 95% CI = 0.634–0.959), heart (AUC = 0.882, 95% CI = 0.768–0.997), lung (AUC = 0.938, 95% CI = 0.842–1.000), and head and neck (AUC = 0.881, 95% CI = 0.754–1.000); *P <* 0.01 (Figure 8, B–N). Next, we calculated the AUC for the traditional inflammatory markers such as C-reactive protein (CRP), D-dimer, neutrophil to lymphocyte ratio (N/L), and troponin to differentiate hospitalized ICU and non-ICU patients (Supplemental Figure 2). We found lower performance compared with the cfDNA profile, with AUC of 0.582 (95% CI = 0.359–0.805; *P =* 0.4611), 0.689 (95% CI = 0.480–0.894; *P =* 0.099), 0.764 (95% CI = 0.567–0.959; *P =* 0.0177), and 0.763 (95% CI = 0.550–0.976; *P =* 0.0343), respectively. Furthermore, we investigated whether cfDNA profile could distinguish hospitalized patients who develop moderate/severe COVID-19 from nonhospitalized patients with mild COVID-19. We found cfDNA levels to have excellent discriminative capacity, with AUC ranging from 0.982 to 0.881 (Supplemental Figure 3, A–P).

### Correlation between cfDNA profiles and measures of end-organ injury.

**Figure 9:**
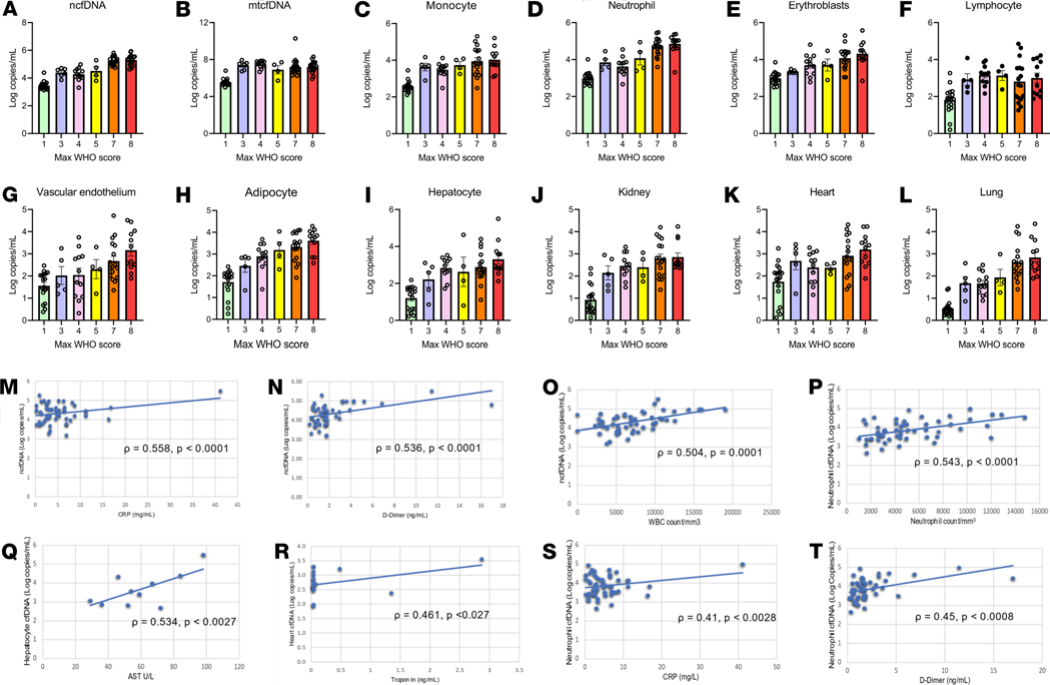
Correlation of cfDNA with WHO scale for COVID-19 severity, inflammatory markers, and markers of tissue injury. Trends of cfDNA profile across WHO ordinal scale 1–8 (1, light green; 3, light blue; 4, light pink; 5, yellow; 7, orange; 8, red): (**A**) ncfDNA, (**B**) mtcfDNA, (**C**) monocyte-derived cfDNA, (**D**) neutrophil-derived cfDNA, (**E**) erythroblast-derived cfDNA, (**F**) lymphocyte-derived cfDNA, (**G**) vascular endothelium–derived cfDNA, (**H**) adipocyte-derived cfDNA, (**I**) hepatocyte-derived cfDNA, (**J**) kidney-derived cfDNA, (**K**) heart-derived cfDNA, and (**L**) lung-derived cfDNA. Correlation between (**M**) ncfDNA and C-reactive protein (CRP), (**N**) ncfDNA and D-dimer, (**O**) ncfDNA and absolute WBC count, (**P**) neutrophil-derived cfDNA and absolute neutrophil count, (**Q**) hepatocyte-derived cfDNA and aspartate aminotransferase (AST), (**R**) heart-derived cfDNA and troponin, (**S**) neutrophil-derived cfDNA and CRP, and (**T**) neutrophil-derived ncfDNA and D-dimer. Spearman’s correlation coefficient was used to calculate statistical significance. *P* values less than 0.05 were considered statistically significant.

The maximum WHO score is one of the most commonly used measures to assess the clinical spectrum of COVID-19. Strikingly, we found a sharp increase in ncfDNA level as the maximum WHO scale ranged from 1 to 8 (Figure 9A). The tissue-specific cfDNA profile from neutrophils, erythroblasts, vascular endothelium, adipocytes, and the lung also showed similar trends. Although an increasing trend was not observed in hepatocyte-, kidney-, and heart-derived cfDNA, we found a higher level for a WHO clinical score of 8 compared with 7. The mtcfDNA and cfDNA level derived from monocytes and lymphocytes was not correlated with the maximum WHO score (Figure 9, B–L).

We then compared cfDNA profiles with known biomarkers of end-organ injury or systemic inflammation and found a significant positive association between ncfDNA and CRP (ρ = 0.558, *P <* 0.0001), D-dimer (ρ = 0.536, *P <* 0.0001), and white blood cell count (ρ = 0.504, *P =* 0.0001). In addition, cell- and tissue-derived cfDNA were associated with organ injury: neutrophil-derived cfDNA and absolute neutrophil count (ρ = 0.543, *P <* 0.0001), hepatocyte-derived cfDNA and aspartate transaminase (ρ = 0.534, *P <* 0.0027), cardiac myocyte–derived cfDNA, and troponin (ρ = 0.461, *P <* 0.027) (Figure 9, M–P). Interestingly, neutrophil-derived cfDNA was also positively correlated with CRP and D-dimer (ρ = 0.41, *P <* 0.0028 and ρ = 0.45, *P <* 0.0008, respectively; Figure 9, Q–T). Thus, tissue-specific cfDNA correlated with traditional measures of inflammation and organ injury.

### COVID-19 plasma induces mitochondrial superoxide production in renal tubular cells.

In a mouse sepsis AKI model, we previously showed that circulating cfDNA, a DAMP, increased mitochondrial superoxide (mtROS) production via a TLR9-dependent inflammatory response (30). So, we evaluated whether COVID-19 patient plasma also contained DAMPs that induce excess mtROS (MitoSOX Red fluorescence measured by confocal microscopy) in mouse primary proximal tubular cells (mPPTCs). To avoid complement activation or oxidative damage from cell debris, plasma was heat-inactivated and centrifuged before being added to cells.

**Figure 10:**
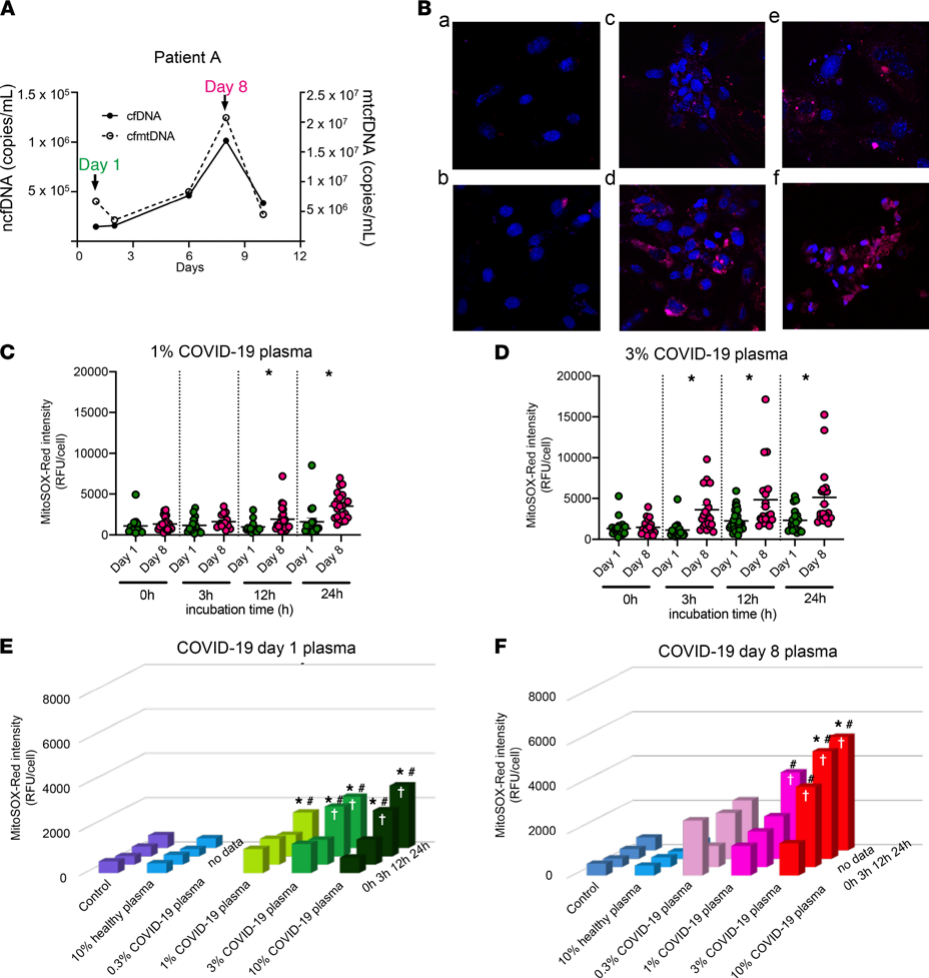
Time- and concentration-dependent generation of mitochondrial ROS. Cell culture of mouse primary proximal tubular cells (mPPTCs) was treated with serial COVID-19 patient plasma. (**A**) mtcfDNA and ncfDNA dynamics of plasma from COVID-19 patient A at day 1 and day 8 after admission. Day 8 plasma contained higher ncfDNA and mtcfDNA than day 1. (**B**) Representative images of mitochondrial superoxide in mPPTCs treated with control (no treatment), 3% healthy volunteer plasma, 1% and 3% COVID-19 patient A’s plasma of day 8 after 3 and 12 hours of incubation: a. Control (no treatment) at 24 hours; b. 24 hours after adding healthy volunteer plasma (3%); c. 3 hours after adding day 8 COVID-19 patient A plasma (1%); d. 3 hours after adding day 8 COVID-19 patient A plasma (3%); e. 12 hours after adding day 8 COVID-19 patient A plasma (1%); f. 12 hours after adding day 8 COVID-19 patient A plasma (3%). Red: MitoSOX Red representing mitochondrial superoxide, blue: Hoechst3342 representing nuclei. Original magnification: ×400. (**C** and **D**) Comparison of MitoSOX Red intensity in mPPTCs (*n =* 18–29 cells from 3–5 fields/group) with (**C**) 1% and (**D**) 3% serial COVID-19 patient A plasma and after 0, 3, 12, and 24 hours of incubation. Each point represents total MitoSOX Red background-corrected intensity for 1 tubule cell. The bar in scatter plots is expressed as mean ± SEM. Statistical significance was determined using a Kruskal-Wallis test and a post hoc Mann-Whitney test. *P* value less than 0.05 considered statistically significant; *P < 0.05: day 1 versus day 8. (**E**–**F**) Incubation time (0, 3, 12, and 24 hours) and plasma dose (0.3%, 1%, 3%, and 10%) dependency of MitoSOX Red intensity in mPPTCs (*n =* 18–29 cells/3–5 fields/group). The 3D bar graph is expressed as mean. Statistical significance was determined using Tukey’s multiple-comparison test after 2-way ANOVA. *P < 0.05 versus control, #*P <* 0.05 versus 3% healthy plasma, †*P <* 0.05 versus 0 hours of incubation.

We applied 1%, 3%, or 10% plasma from COVID-19 patient A (Figure 10A) onto mPPTCs and then captured longitudinal images at 0, 3, 12, and 24 hours (Figure 10B). Both day 1 and day 8 plasma increased superoxide in a dose- and time-dependent manner compared with untreated or 3% plasma from a healthy volunteer (Figure 10, E and F). Moreover, day 8 plasma increased MitoSOX Red intensity more than day 1 plasma (Figure 10, C and D); day 8 plasma showed higher cfDNA than day 1 plasma. Similar results were seen in patient B (Supplemental Figure 4).

### COVID-19 cfDNA induces mtROS production, which is attenuated by TLR9 inhibition.

**Figure 11:**
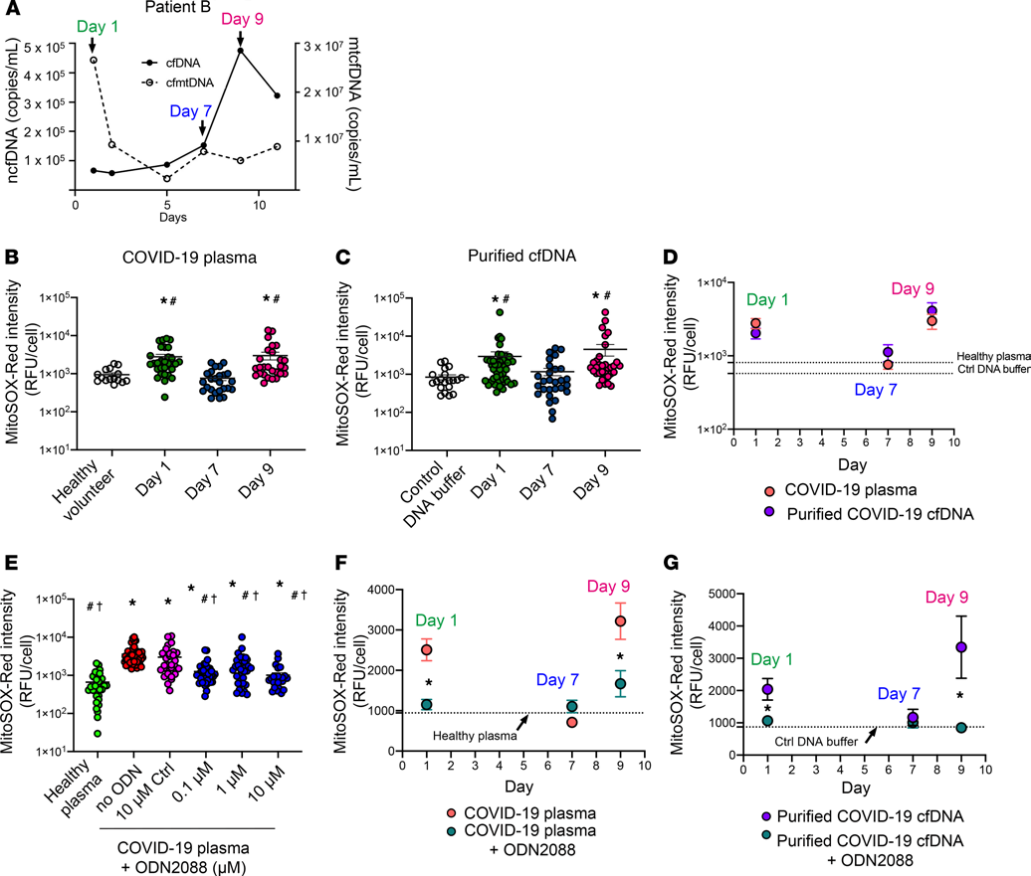
Purified plasma cfDNA stimulation of mtROS production and the effect of TLR9 inhibition. mPPTC culture was treated with plasma of COVID-19 patient B or purified cfDNA from the same plasma samples. (**A**) mtcfDNA and ncfDNA dynamics in plasma from COVID-19 patient B. (**B**) mPPTCs (*n =* 15–44 cells from 3–5 fields/group) treated with 3% healthy plasma or serial 3% COVID-19 patient B plasma and (**C**) with control DNA buffer and equivalent amount of cfDNA purified from serial COVID-19 patient B plasma; MitoSOX Red intensity measured after 6 hours incubation. Data summarized as in Figure 9. Statistical significance determined using Dunnett’s multiple-comparison test after Kruskal-Wallis test. *P < 0.05 versus control, #*P <* 0.05 versus day 7. (**D**) Comparison of MitoSOX Red intensity with serial COVID-19 patient B plasma (red) and cfDNA purified from COVID-19 patient B plasma (purple) after 6 hours incubation. Each point represents total MitoSOX Red background-corrected intensity for 1 tubule cell. Line graph expressed as mean ± SEM. Data analyzed using Mann-Whitney test after Kruskal-Wallis test. (**E**) MitoSOX Red intensity in mPPTCs (*n =* 26–47 cells from 3–6 fields/ group) treated with 3% healthy plasma; 3% COVID-19 patient plasma with 10 μM control oligodeoxynucleotide (ODN); or 0, 0.1, 1, or 10 μM TLR9 antagonist (ODN2088) after 6 hours incubation. Statistical significance determined using Dunnett’s multiple-comparison test after Kruskal-Wallis test. *P < 0.05 versus 3% healthy plasma, #*P <* 0.05 versus no ODN, †*P <* 0.05 versus 10 μM control ODN. (**F**) Comparison of MitoSOX Red intensity in mPPTCs (*n =* 17–63 cells from 3–6 fields/group) treated with serial COVID-19 plasma or COVID-19 plasma with ODN2088, or (**G**) serial cfDNA purified from the COVID-19 patient B plasma with and without ODN2088 after 6 hours incubation. Statistical significance determined using a Mann-Whitney test after Kruskal-Wallis test. *P < 0.05. mtROS production of patient B shown in Supplemental Figure 4.

Next, we evaluated whether cfDNA contained in COVID-19 plasma could generate mtROS in mPPTCs. We purified cfDNA from COVID-19 patient plasma, then added an amount of cfDNA that was equivalent to 3% plasma into mPPTCs. We tested serially collected plasma from COVID-19 patient B because the ratio of ncfDNA to mtcfDNA changed dramatically over time (day 1: mtcfDNA-rich/ncfDNA-poor cfDNA; day 7: mtDNA- and nDNA-poor cfDNA; day 9: mtcfDNA-poor/ncfDNA-rich cfDNA) (Figure 11A). Interestingly, both day 1 and day 9 plasma increased MitoSOX Red fluorescence, but day 7 plasma did not increase MitoSOX Red fluorescence, compared with healthy control plasma (Figure 11B). cfDNA purified from both day 1 (mtcfDNA-rich) and day 9 (ncfDNA-rich) plasma increased superoxide compared with day 7 cfDNA (mtDNA and nDNA both low) and control DNA buffer (Figure 11C). When purified cfDNA was compared with an equivalent volume of plasma, there was no difference in the production of superoxide on days 1, 7, and 9 (Figure 11D).

Next, we tested whether a TLR9-specific antagonist could inhibit overproduction of mtROS induced by COVID-19 plasma or purified cfDNA (positive control). CpG-oligonucleotide (CpG-ODN) is a TLR9 ligand, and ODN2088 is a mouse TLR9-specific antagonist that inhibits the stimulatory effect of CpG ODNs. First, we tested the ability of increasing concentrations of ODN2088 to inhibit the effect of 3% COVID-19 patient plasma on MitoSOX Red fluorescence. At every concentration tested, ODN2088 inhibited the COVID-19 plasma increase in MitoSOX Red fluorescence versus controls (Figure 11E); 10 μM ODN2088 was more effective than 1 μM ODN2088. Next, we evaluated the inhibitory effect of ODN2088 on mtROS generation by cfDNA purified from COVID-19 patient B. We found 10 μM ODN inhibited MitoSOX Red fluorescence generated by day 1 and day 9 cfDNA (Figure 11G) to the same extent as day 1 and day 9 plasma (Figure 11F). We conclude that most of the COVID plasma effect on superoxide production requires cfDNA activity via the TLR9 receptor; however, we cannot rule out whether other mediators are also involved, considering that ODN 2088 can affect other pathways (36).

## Discussion

This study expands the use of cfDNA from being a potential diagnostic biomarker that maps sources of tissue injury to being a prognostic biomarker that predicts COVID-19 trajectory and outcome and to providing mechanistic information about COVID-19–induced tissue injury. We found markedly elevated levels of total (bulk) plasma cfDNA in patients with COVID-19, including both nuclear and mitochondrial origin; cfDNA predominantly originated from hematopoietic cells. Notably, the cfDNA profiles in patients infected with other respiratory viruses, influenza and RSV, were significantly lower and divergent in origin from that of patients with COVID19. Patients with COVID-19 also showed divergent tissue sources of cfDNA as early as in the first few days of admission, including cfDNA from neutrophils, adipocytes, cardiac myocytes, and vascular endothelial cells. Tissue-specific cfDNA markers correlated with known biochemical measures of end-organ injury. Some of these tissue-specific cfDNA markers demonstrated high performance to identify patients who required ICU care or who subsequently died, similar to the findings of a recent report (37). After multivariate analysis correcting for known risk factors for severe COVID-19 disease, including increasing age, male sex, and obesity, there remained a significant association of elevated risk of poor clinical outcome. Isolated cfDNA, which contains both cfDNA and mtcfDNA, orchestrated a TLR9-dependent stimulation of mitochondrial ROS that may set up a vicious cycle of tissue injury.

This methylation deconvolution algorithm captured cfDNA from multiple tissue types and showed a divergent cfDNA tissue source in COVID-19 patients compared with healthy controls or some common respiratory viruses such as influenza and RSV. After matching for disease severity, COVID-19 patients showed higher ncfDNA, as well as cfDNA from adipocytes, vascular endothelial cells, the pancreas, cardiac myocytes, and the kidney compared with influenza and RSV patients. Within COVID-19 patients, this approach showed different tissue sources of cfDNA, representing different tissue injury patterns. These findings indicate that COVID-19 infection orchestrates widespread tissue injury, with predilection for some tissue types, as has been directly demonstrated in autopsy studies (alveolar damage, coagulopathy, kidney injury, and cardiac injury) (6, 38–40). This cfDNA approach is potentially informative to catalog the tissue sources of injury and organ dysfunction for any unknown condition, including new strains of SARS-CoV-2, unknown autoimmune conditions, or other diseases.

The cfDNA profiles in patients with COVID-19 correlated with disease severity, assessed by WHO ordinal scale, a 1 to 8 scale commonly used to stratify patients by COVID-19 severity. Interestingly, cfDNA measurements at admission correlated with the subsequent disease trajectory measured as the maximum WHO scale during patients’ COVID-19 illness. Similar findings have been reported for mtcfDNA (41). For example, as early as days 0 to 2 of admission, cfDNA levels could be used to identify patients who required ICU care (WHO scale 5–8) or died (WHO scale 8) during their illness. Similarly, hospitalized patients not requiring ICU care (WHO scale 3–4) showed higher cfDNA profiles compared with patients with mild COVID-19 not requiring hospitalization (WHO scale 1–2). Interestingly, some tissue-specific cfDNA markers such as cardiac cfDNA were increased only in patients with COVID-19 with the highest score on the WHO scale. A previous study confirmed that ncfDNA, but not mtcfDNA, directly contributed to cellular death and predicted clinical trajectories in the setting of severe trauma (42). Neutrophil-to-lymphocyte ratio and systemic inflammatory markers like CRP have been used as an early predictor of COVID-19 severity (43, 44). Our results indicate cfDNA profiles on admission can discriminate between patients with COVID-19 at risk of severe disease and death with better performance than previously reported inflammatory markers.

cfDNA measures correlated with known biomarkers of severe disease or tissue injury. For example, absolute neutrophil count was strongly correlated with neutrophil-derived cfDNA. We also showed a strong positive correlation between inflammatory biomarkers such as CRP and D-dimer to total ncfDNA and neutrophil-derived cfDNA. This suggests that neutrophils might be involved in aggravating inflammation and is consistent with studies that show an increase in nonspecific acute-phase CRP and D-dimer inflammatory biomarkers in COVID-19, particularly in patients who subsequently have poor outcomes (45, 46). Finally, tissue-specific cfDNA correlated with biochemical markers of tissue injury; for example, cardiac myocyte–specific cfDNA to troponin- or hepatocyte-specific cfDNA to liver function markers such as aspartate transaminase.

Hematopoietic cells remain a major contributor, contributing a greater fraction of cfDNA in COVID-19 patients than in healthy controls, particularly neutrophil-derived cfDNA. Interestingly, neutrophils were the major contributor to an elevated cfDNA level in COVID-19 patients, particularly in the patients who required ICU care or who subsequently died. Although neutrophils are primarily involved in early antiviral defense (47), they can aggravate pulmonary inflammation. Indeed, recent studies reported neutrophil infiltration into the lung (48) and generation of NETs, which then promote excessive inflammation and intravascular thrombosis that results in damage of multiple tissues/organs in severe COVID-19 (49, 50). Patients with COVID-19 also exhibited aberrations in erythropoiesis, with increasing nucleated red blood cells in the circulation (51). We found erythroid precursors as the second most predominant contributor of hematopoietic origin, suggesting increased erythroblast turnover. Nonhematopoietic tissue types also released cfDNA at levels that were significantly higher than in healthy controls. Among the nonhematopoietic tissues, we detected significantly increased cfDNA derived from the vascular endothelium, adipocytes, liver, pancreas, intestine, bladder, kidney, heart, and lung in COVID-19 patients compared with healthy controls. These results are consistent with clinical presentations of patients with COVID-19 (52–55) and reflect the involvement of multiple cells/tissues/organs in COVID-19 pathogenesis.

Elevated levels of circulating cfDNA released from injured tissue have been associated with worse clinical outcomes in multiple settings, including trauma (42), sepsis (56), autoimmunity (57), and transplantation (12). In addition to being a useful biomarker, cfDNA could play a pathogenic role. cfDNA are DAMPs that trigger inflammation (58), thereby adding an additional mechanism for tissue injury. Herein, we found that the excessive ncfDNA and mtcfDNA in COVID-19 plasma caused the overproduction of mtROS in kidney tubule cells via TLR9, which might contribute to a positive feedback loop that contributes to the pathogenesis of COVID-19 multiple organ failure; that is, tissue injury leads to the release of cfDNA, which then acts on other tissue types via TLR9 to trigger additional tissue injury. As with these studies, we cannot distinguish between ncfDNA and mtcfDNA without additional purification. Because of the unclear nature of TLR9 binding sites, it is also not clear whether the copy number or mass is more relevant, making direct comparisons between mtcfDNA and ncfDNA difficult.

AKI develops in 20% to 33% of patients with COVID-19 (59, 60), generally in patients with more severe infection, and portends a higher mortality rate (61). Acute tubular injury is a common pathological finding in COVID-19–induced AKI and polymicrobial septic AKI (62). TLR9 activates an innate immune response to produce proinflammatory cytokines via an MyD88-dependent pathway (63, 64). During polymicrobial sepsis, mtcfDNA activates TLR9 and contributes to cytokine production, splenic apoptosis, and tubular injury with mtROS overproduction (30). Inhibition of TLR9 or MyD88 attenuates septic AKI and improves survival (30, 65, 66).

Although cfDNA is a promising biomarker, cfDNA has a limitation of intra- and interindividual variability, from day to day and within days; thus, cfDNA kinetics and reference interval must be well evaluated (67). The deconvolution algorithm includes DNA methylation signatures of the predominant cell in each tissue type; for example, cardiac myocyte cfDNA from the heart or hepatocyte cfDNA from the liver. As such, the algorithm does not capture cfDNA from less predominant cell-types of tissue, which may also show clinical relevance. Further, the deconvolution algorithm captures cfDNA from a wide range, but not all tissue types. Nonetheless, we showed that the cfDNA profiles captured were clinically relevant. Additional limitations of this study include the missing clinical data and laboratory measures for some patients and the lack of serial plasma sampling in a small number of study participants. Future studies are warranted to validate these findings in a larger cohort by dissecting clinical phenotypes to test the applicability of the technology and investigate the role of cfDNA as a trigger of tissue injury in other organs.

Taken together, these findings demonstrated that plasma cfDNA provides a comprehensive profile of tissue injury in patients with COVID-19. The levels (both bulk plasma and tissue-specific) provide useful prognostic biomarkers for patient trajectory and morbidity. These markers may be useful to identify high-risk patients early in the course of COVID-19 to initiate useful treatment. The cfDNA is also a DAMP, resulting in the overproduction of ROS, which causes excessive inflammation and collateral tissue damage. If validated, cfDNA will be used as a noninvasive clinical tool, and inhibition of mtROS and/or TLR9 signaling are potential therapeutic targets.

## Methods

### Study design, setting, and participants.

We conducted a prospective cohort study at Johns Hopkins Hospital, University of Maryland Medical Center, and Inova Fairfax Hospital from April 21, 2020, to September 30, 2020. At each institution, patients diagnosed with COVID-19 by positive SARS-CoV-2 RNA testing were enrolled in a protocol designed to generate a biospecimen repository linked to clinical data for investigation. Participants identified as SARS-CoV-2 PCR positive consented to study participation and for clinical information to be linked to their study subject identification number. Samples, including blood for processing into serum, plasma, and PBMCs, were obtained closest to admission time (day 0), a few times per week thereafter, and weekly after day 7 until discharge for hospitalized patients. For nonhospitalized COVID-19 patients, a single time-point sample was collected close to the day of their positive test. Demographic information, clinical laboratory test results, ICD-10–coded diagnoses recorded in the patients’ records (comorbidities), medication lists, body mass index, and other clinical parameters were linked to data for all participants in the study. The study also collected plasma from 32 healthy controls and 21 patients who tested positive for influenza and/or RSV to serve as a comparator group. Samples from patients with influenza and/or RSV were collected on the day of their positive test, which was on admission for hospitalized patients or at clinic visits for nonhospitalized patients. Demographic data, clinical characteristics, health care utilization data, and laboratory assessment of end-organ injury were collected. The WHO 8-point ordinal scale measurement for clinical improvement, which is based on health care resource utilization and death (35) was used to stratify patients with COVID-19 by disease severity into 3 groups: 1) Nonhospitalized mild cases (WHO scale 1, 2), 2) hospitalized non-ICU moderate cases (WHO scale 3, 4) and 3) hospitalized ICU severe cases (WHO scale 5–8). The study participant selection and experiment are summarized in Figure 1. The primary analyses assessed whether cfDNA parameters measured early in the disease course (patient admission/test) could identify hospitalized patients who died or required ICU care; day 0 to 2 of admission or positive test result were selected arbitrarily to represent early COVID-19 disease. We also compared total cfDNA and tissue-specific cfDNA measures between groups to identify differences in cfDNA trends. Participants were also stratified based on their maximum WHO score to assess cfDNA measures in relation to the need for ICU (WHO scale 3–4 vs. 5–8) or death (WHO scale 3–7 vs. 8).

### Plasma sample collection.

Whole blood was drawn into a cfDNA blood collection tube (Streck), centrifuged at 1600*g* for 10 minutes at 4°C to separate plasma, and stored immediately at –80˚C. At 2 centers, whole blood was collected in EDTA tubes and centrifuged within 1 to 2 hours before significant cell lysis occurred and stored at –80˚C until used. For cfDNA isolation, 1 mL of plasma was used as starting material. The sample was centrifuged at 4°C at 16,000*g* for 5 minutes to remove residual debris and spiked with 0.142 ng of 170 bp–fragmented unmethylated lambda DNA (Promega, D1521) to measure the extraction efficiency and bisulfite conversion efficiency. The plasma samples were diluted with 1 mL of PBS prior to cfDNA isolation by QIAsymphony circulating DNA kit using a customized 2 mL protocol. The cfDNA was eluted in 60 μL of low-EDTA Tris-EDTA (TE) buffer.

### cfDNA quantification.

The isolated cfDNA was quantified by measuring copies of both ncfDNA and mtcfDNA with digital droplet PCR (ddPCR) copy number determination assays (Bio-Rad assay ID: dHsaCP2500316 and dHsaCNS669425578) on a QX200 ddPCR system. One assay is targeted to eukaryotic translation initiation factor 2C1 (EIF2C1) for ncfDNA quantification and another one is targeted to NADH dehydrogenase 1 (ND1) for mtcfDNA. Briefly, a total volume of 22 μL ddPCR reaction mixture containing 11 μL 2× ddPCR Supermix for Probes (No dUTPs), 4 μL template DNA (10- to 100-fold diluted), and 0.55 μL of each 20× ddPCR assay and 4.8 μL nuclease-free water were partitioned into approximately 17,000 droplets on Bio-Rad Automated Droplet Generator, in triplicate. Droplet range was 13,200–22,500 in our experiments, which was higher than the manufacturer’s limit of 10,000 droplets per sample. The droplets generated were amplified with the following thermal conditions: 95°C for 10 minutes, followed by 40 cycles of 15 seconds at 95 °C and 1 minute at 60°C with ramp rate set to 2.5°C/s. Finally, droplets were read on Q×200 droplet reader and data were analyzed using QuantaSoft software.

The spiked lambda DNA fragments were quantified by qPCR. Briefly, a 10 μL PCR mixture containing 2 μL cfDNA template (1:10 diluted), 5 μL SYBR Green Supermix (Bio-Rad), 2 μL nuclease-free water, and 1 μL primer pair were prepared in triplicate. The reaction mixture was run on QuantStudio 3 qPCR cycler (Applied Biosystems, Thermo Fisher Scientific), with an initial denaturation of 95°C for 5 minutes followed by 35 cycles of 95°C for 15 seconds and annealing at 60°C for 1 minute. The concentrations were calculated using a standard curve generated from 10-fold serially diluted (1.1 ng to 1.1 × 10–5 ng) lambda DNA (Promega). The primer sequences were as follows: forward, 5′-GACCTCTATGCCAACACAGT-3′ and reverse, 5′-AGTACTTGCGCTCAGGAGGA-3′. The extraction efficiency was calculated by dividing the lambda DNA detected in qPCR by the original concentration of lambda DNA spiked into the plasma.

### Bisulfite treatment, library construction, validation, and sequencing.

The quality of extracted cfDNA was analyzed by cfDNA ScreenTape analysis on the 4150 TapeStation System (Agilent Technologies) prior to bisulfite conversion. The cfDNA was then processed with EZ DNA methylation-gold kit (Zymo Research) as per the manufacturer’s recommendation to convert unmethylated cytosine residues to uracil. The methyl-Seq DNA library was prepared using the Accel-NGS Methyl-Seq DNA Library Kit with Unique Dual Indexing (Swift Biosciences) for whole-genome bisulfite sequencing. The quality of the constructed DNA library was assessed using a high-sensitivity D1000 ScreenTape and quantified by the Quant-iT PicoGreen dsDNA assay kit (Life Technologies). The DNA libraries were then pooled in equimolar concentrations and subjected to 2 × 100 bp paired-end DNA sequencing on the Illumina NovaSeq 6000 platform.

### Bioinformatics analysis.

Sequence reads were analyzed as follows: quality control, trimming, and mapping to the human reference sequence (hg38 assembly) with FastQC (https://www.bioinformatics.babraham.ac.uk/projects/fastqc/), TrimGalore (http://www.bioinformatics.babraham.ac.uk/projects/trim_galore/) (68), and Bismark (69), respectively; 10 bp were trimmed from both ends of read1 and read2, retaining paired-end reads with a minimum length of 50 bp; PCR duplicate removal and postalignment quality control were also performed using Bismark. The Bismark methylation extractor routine determines cytosine methylation states and extracts all CpGs in individual samples. The custom-built analytic workflow then uses a collection of tools from bsseq (70) for analyzing and visualizing the cf-methylome data.

We used the meth_atlas algorithm (15) to identify the tissue or cell type of origin of cfDNA and deconvolution of the cfDNA methylome by sample. Meth_atlas uses a reference methylation atlas of 25 human tissues and cell types to determine the tissue origins of cfDNA. It covers major organs and cells involved in common diseases. Using in silico simulations as well as in vitro mixes of DNA from different tissue sources at known proportions, it has been shown to effectively identify cfDNA tissue of origin using a small number of loci (~4,000 CpGs; ref. 15). It approximates the plasma cfDNA methylation profile as a linear combination of the methylation profiles of cell types in the reference atlas. To obtain the concentrations of cfDNA in the tissue or cell type of origin, the relative estimated proportions of cfDNA were multiplied by the total concentration (or copies per mL) of cfDNA in plasma and adjusted with the extraction efficiency. The sum of individual cfDNA from individual tissue types should equal total ncfDNA. However, we represented only the most predominant tissue sources of cfDNA. We used CpGs with at least 5× coverage per sample for the deconvolution analysis and analysis was performed using R software version 3.6.3. The deconvolution algorithm scripts are at https://github.com/nloyfer/meth_atlas and the methylation analysis scripts are at https://github.com/seifudd/cfMethylome

### Cell preparation.

mPPTCs were prepared from CD-1 mice as reported previously (71). In brief, kidneys were harvested, decapsulated, cut into small pieces with sterile instruments, and digested in tissue collagenase type I (30 mg/g; MilliporeSigma) for 30 minutes at 37°C. The material was pushed through a 70 μm sieve (BD Biosciences), washed, and diluted in 2 mL PBS. The tubular segments were separated by 31% Percoll (GE Healthcare) centrifugation at 800 *g* for 10 minutes at 4°C. The pellet was collected and washed twice with PBS at 370 *g* for 5 minutes at 4°C. The isolated mPPTCs were cultured under sterile conditions at 37°C and 5% CO_2_ in conditioned Renal Epithelial Cell Growth Medium 2 (PromoCell). mPPTCs isolated by this method have been reported to be mainly proximal tubule epithelial cells (72).

### Live-cell imaging of mtROS.

mPPTCs were plated onto a chambered cover glass slip (Cellvis), incubated with optimized medium for 18–24 hours, stained with 200 nM MitoSOX Red and 2 μg/mL Hoechst 33343 (Thermo Fisher Scientific) for 30 minutes at 37°C, washed with HBSS with Ca and Mg, and then incubated with ProLong Live Antifade Reagent (Thermo Fisher Scientific). COVID-19 patient plasma was heated at 56°C for 30 minutes to inactivate complement, and then centrifuged at 16,000*g*, 4°C for 10 minutes. Plasma from COVID-19 patients, or the equivalent amount of cfDNA purified from the plasma, was added to the chamber. Some chambers were treated with a TLR9-specific antagonist (ODN2088) or negative control for ODN2088 (control ODN2088; InvivoGen). Images were acquired at several points until 24 hours after incubation by confocal microscopy outfitted with an environmental incubation system (Zeiss LSM780). MitoSOX Red and Hoechst 33343 fluorescence were imaged at 488 nm and 405 nm excitation and using 562–666 nm and 413–490 nm emission ranges, respectively. All imaging parameters/settings remained the same for all data acquisition using Zen Zeiss software. The relative fluorescence of MitoSOX Red in individual cells was quantified using Fiji or ImageJ (NIH) software and adjusted for background fluorescence. Fluorescence intensities were log-transformed to achieve a more approximately normal distribution, but not all groups passed each normality test, and therefore nonparametric post hoc tests were used.

### Statistics.

Data are presented as mean ± SEM for continuous variables unless indicated otherwise and frequency (percentage) for categorical variables. Statistical difference between groups was calculated using an unpaired 2-sided Student’s *t* test for pairwise comparisons assuming unequal variances (i.e., Welch’s test). For multiple comparisons for a given measure among more than 2 groups, Hommel’s procedure (to control overall false-positive rate at the 0.05 level) was used to confirm that conclusions did not differ substantially, as listed in Supplemental Table 3, using the multxpert package within R (version 3.6.2; ref. 73). Comparisons for more than 2 groups were done by using the Kruskal-Wallis rank test for skewed data. Multivariable logistic regression was performed after adjustment for clinical and demographic variables. Variables were included within the multivariable model if they met the threshold for statistical significance in the univariable analysis (*P =* 0.10) and/or if they were deemed clinically relevant. Variables included for comparison that met these criteria were age, sex, obesity, and presence of cancer.

Area under the ROC of cfDNA profile was calculated to assess the discriminative capacity of COVID-19 patients by clinical trajectory (need for ICU: WHO scale 5–8 vs. non-ICU: WHO scale 1–4) and outcome (recovered: WHO scale 1–7 vs. deceased: WHO scale 8). Categorical variables were compared with Fisher’s exact test. Correlations between cfDNA profiles and quantitative clinical markers of tissue damage were done by Spearman’s (ρ) correlation. The comparator group of subjects with influenza and/or RSV was matched to COVID-19 patients using the WHO scale to compare total and tissue-specific cfDNA measures. In the analysis of MitoSOX Red intensity in cells, the data are shown as mean ± SEM. Statistical significance was determined using the Kruskal-Wallis test, followed by the Mann-Whitney test for 2 groups or Tukey’s or Dunnett’s multiple-comparison test. Two-way ANOVA followed by Tukey’s multiple-comparison test was used for comparison of time courses or dose response. Statistical analyses were performed using GraphPad Prism version 9 (and version 3.6.2; *P* values less than 0.05 were considered significant unless otherwise indicated).

### Study approval.

The study was approved by IRBs of the Johns Hopkins University School of Medicine, University of Maryland Medical Center, and Inova Fairfax Hospital. Study subjects provided informed consent for study participation at Johns Hopkins Hospital and Inova Fairfax Hospital. The IRB approved a consent waiver at University of Maryland to use excess samples collected for clinical need.

## Author contributions

TEA, NT, MKJ, PSTY, HK, RAS, and SAE designed experiments. TEA, NT, MKJ, HK, AC, SN, and PSTY acquired data. FS, IT, KS, and MP performed bioinformatics analysis. TEA, NT, and SAE wrote the manuscript. SN and AC recruited patients and provided samples and clinical data. TEA, FS, MP, NT, PSTY, SAE, RAS, and KW performed statistical analysis. All authors participated in preparation of the manuscript and gave final approval for publication.

## Supplementary Material

Supplemental data

Trial reporting checklists

ICMJE disclosure forms

## Figures and Tables

**Table 1:**
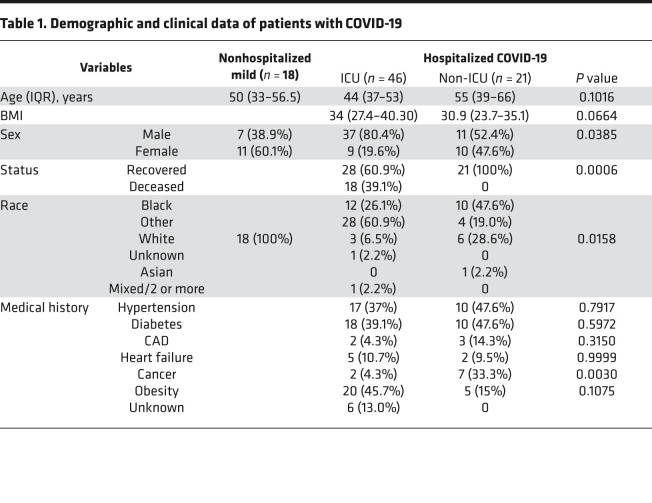
Demographic and clinical data of patients with COVID-19

## Version 1 03/02/2021

In-Press Preview

## Version 2 04/08/2021

Electronic publication
