# Supplementary material for: Cell-free DNA maps COVID-19 tissue injury and risk of death and can cause tissue injury
Source: JCI Insight. 2021 Apr 8;6(7):e147610. doi: 10.1172/jci.insight.147610 (PMC8119224; doi:10.1172/jci.insight.147610)
Supplement: Supplemental data [file jciinsight-6-147610-s260.pdf]

Supplementary Material

Cell-free DNA maps COVID-19 tissue injury and risk of death, and propagates tissue injury

Temesgen E. Andargie[#,1,2], Naoko Tsuji[#,3], Fayaz Seifuddin[4], Moon Kyoo Jang[1], Peter S.T. Yuen[3], Hyesik Kong[1], Ilker Tunc[4], Komudi Singh[4], Ananth Charya[1], Steven Nathan[5], Andrea Cox[6], Mehdi Pirooznia[4], Robert A. Star[3], Sean Agbor-Enoh[*,1,6]

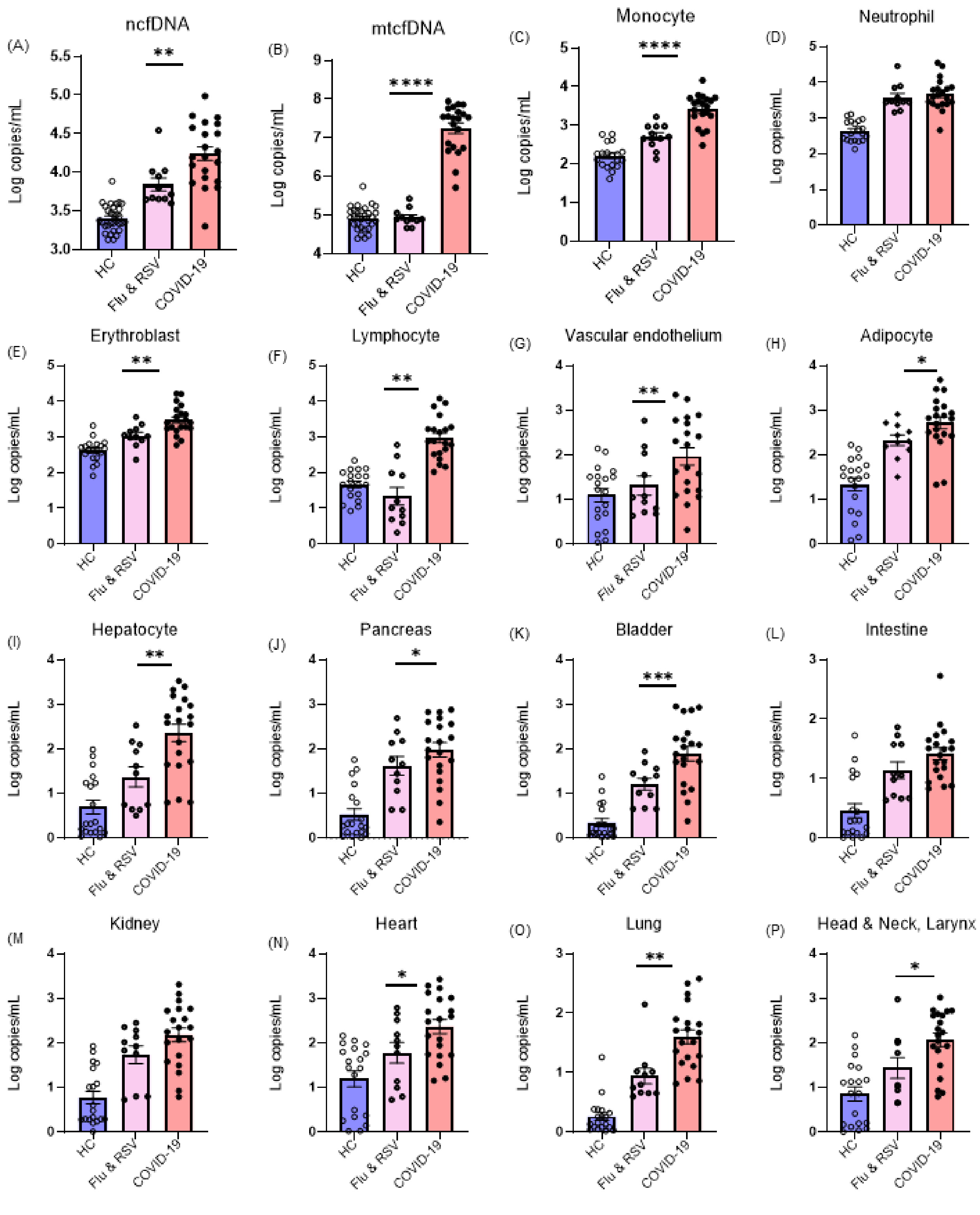

(A) ncfDNA
(B) mtcfDNA
(C) Monocyte
(D) Neutrophil
(E) Erythroblast
(F) Lymphocyte
(G) Vascular endothelium
(H) Adipocyte
(I) Hepatocyte
(J) Pancreas
(K) Bladder
(L) Intestine
(M Kidney
(N) Heart
(O) Lung
(P) Head & Neck, Larynx
Log copies/mL
HC
Flu & RSV
COVID-19

Supplementary Figure 1. cfDNA profiles in COVID-19 patients compared with WHO-scale-matched Flu & RSV patients.

Total plasma cell-free nuclear (A) and mithochondrial cfDNA (B) from Healthy controls, blue (n=31), hospitalized non-ICU Influenza and Respiratory Syncytial Virus (Flu & RSV) infected patients, orange (n=12), and hospitalized non-ICU COVID-19 patients, black (n= 20). (C-P) quantification of cell/tissue-specificf cfDNA; (C) Monocytes, (D) Neutrophils, (E) Erythroblasts, (F) Lymphocytes, (G) Vascular endothelial cells, (H) adipocytes, (I) Hepatocytes, (J) Pancreas, (K) Bladder, (L) intestine, (M) Kidney, (N) Heart, (O) Lung and (P) Head and neck larynx-derived cfDNA from HCs, brown (n=19), Flu & RSV patients, yellow (n=12), and COVID-19 patients, red (n=20).Statistical significances were determined by unpaired 2-sided student's t-test. Bar graphs expressed as mean ± standard error of the mean (SEM). P-values less than 0.05 were considered statistically significant; *p < 0.05, **p < 0.01, ***p < 0.001, and ****p < 0.0001.

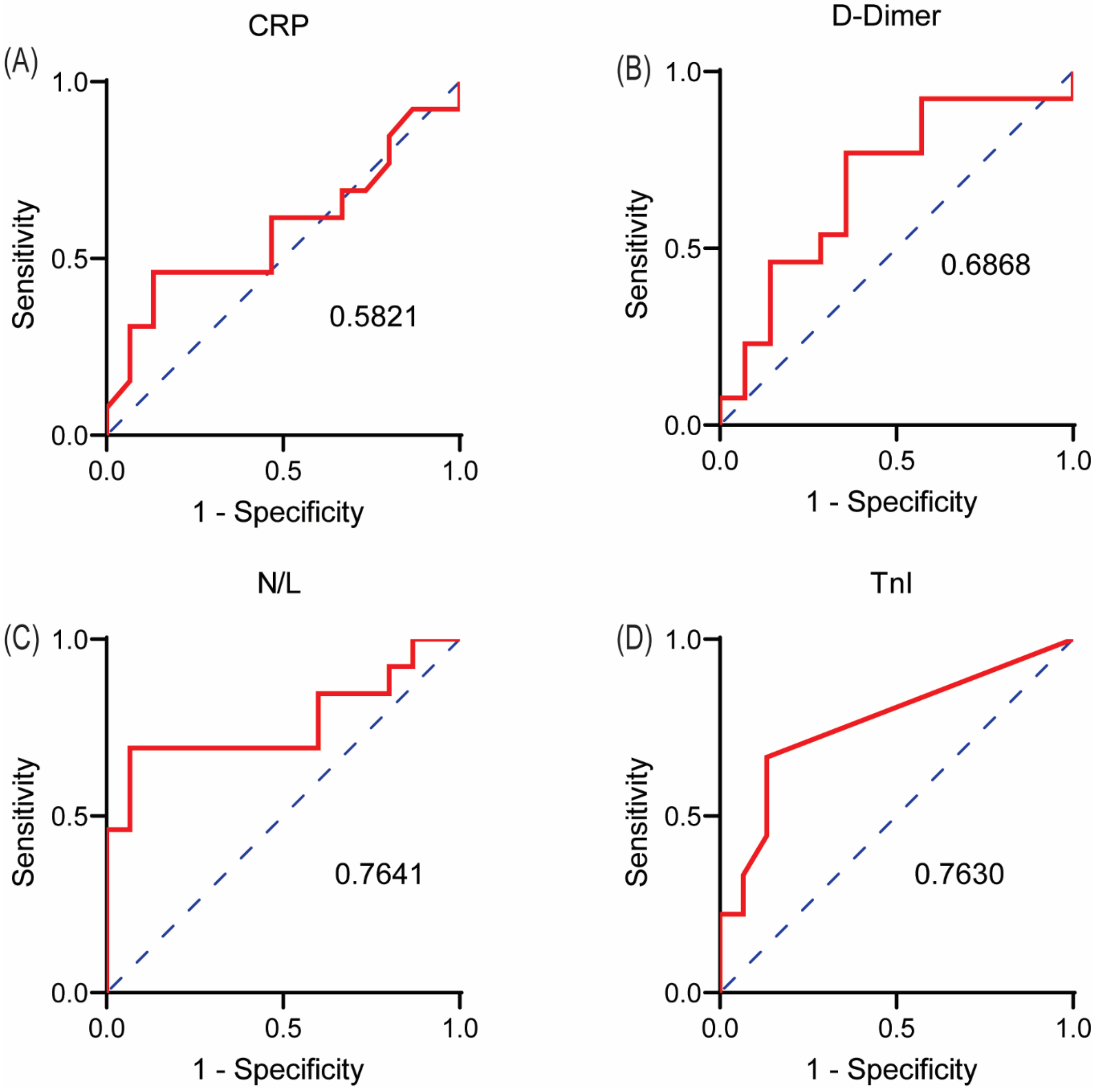
CRP
(A)
1.0
0.5
0.0
Sensitivity
0.5821
0.0
0.5
1.0
1 - Specificity
D-Dimer
(B)
1.0
0.5
0.0
Sensitivity
0.6868
0.0
0.5
1.0
1 - Specificity
N/L
(C)
1.0
0.5
0.0
Sensitivity
0.7641
0.0
0.5
1.0
1 - Specificity
TnI
(D)
1.0
0.5
0.0
Sensitivity
0.7630
0.0
0.5
1.0
1 - Specificity

Supplementary Figure 2. ROC analysis showing performance traditional inflammatory markers to identify hospitalised patients who subsequently require ICU versus who did not: A) CRP, (B) D-Dimer, (C) Neutrophil to Lymphocyte ration (N/L) and (D) Troponin.

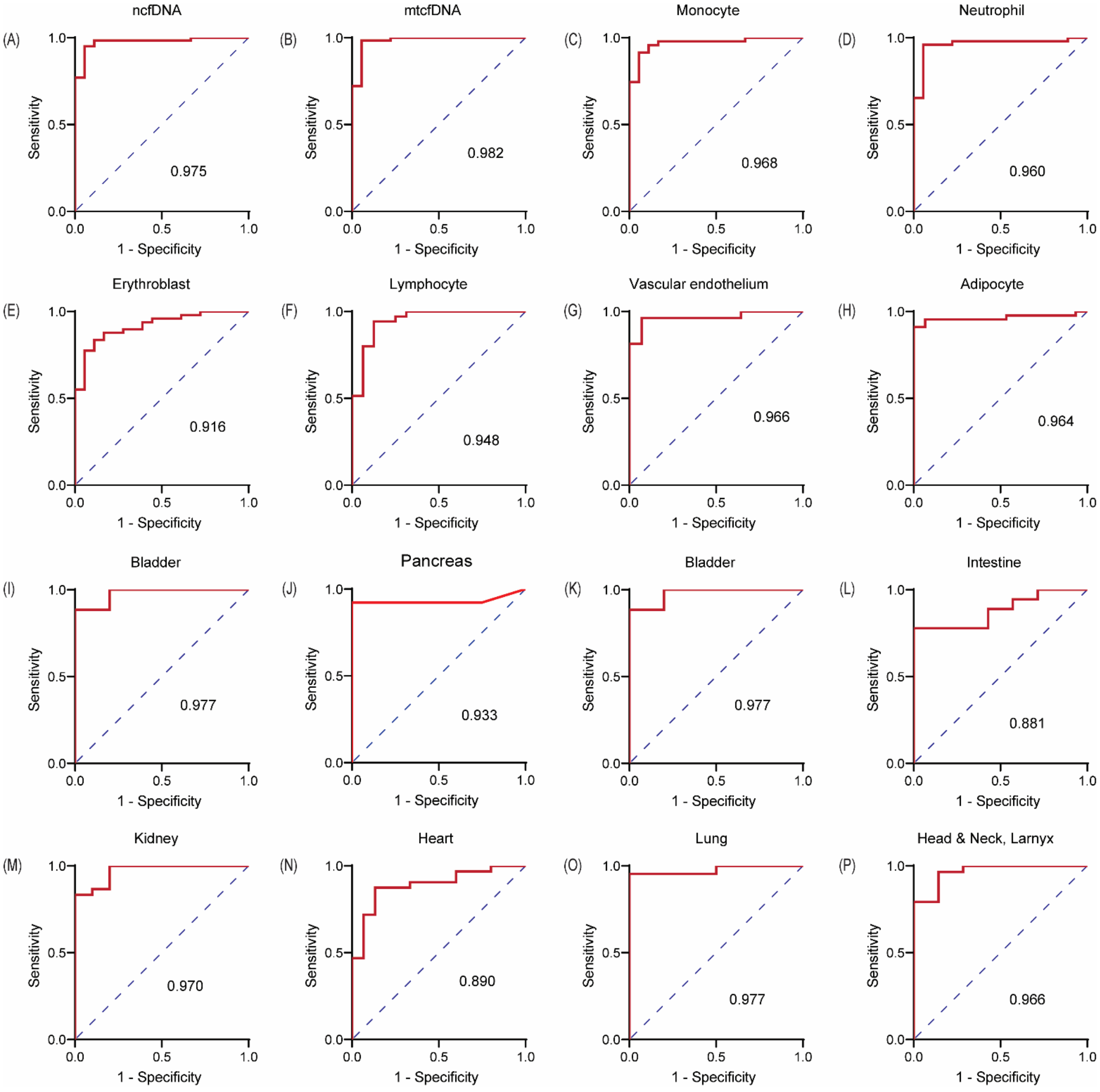

ncfDNA
mtcfDNA
Monocyte
Neutrophil
(A)
(B)
(C)
(D)
0.975
0.982
0.968
0.960
Erythroblast
Lymphocyte
Vascular endothelium
Adipocyte
(E)
(F)
(G)
(H)
0.916
0.948
0.966
0.964
Bladder
Pancreas
Bladder
Intestine
(I)
(J)
(K)
(L)
0.977
0.933
0.977
0.881
Kidney
Heart
Lung
Head & Neck, Larnyx
(M)
(N)
(O)
(P)
0.970
0.890
0.977
0.966
Sensitivity
1 - Specificity
0.0
0.5
1.0

Supplementary Figure 3. ROC analysis testing the performance of cfDNA to distinguished hospitalized patients from patients with mild COVID-19 not requiring hospitalization: (A)ncfDNA, (B) mtcfDNA , (C) Monocyte-, (D) Neutrophil-, (E) Erythroblast-, (F) Lymphocytes, (G) Vascular endothelium- (H) adipocyte-, (I) Hepatocyte-, (J) Pancreas, (K) Bladder-, (L) intestine-, (M) Kidney-, (N) Heart-, (O) Lung- and (P) Head and neck larynx-derived cfDNA.

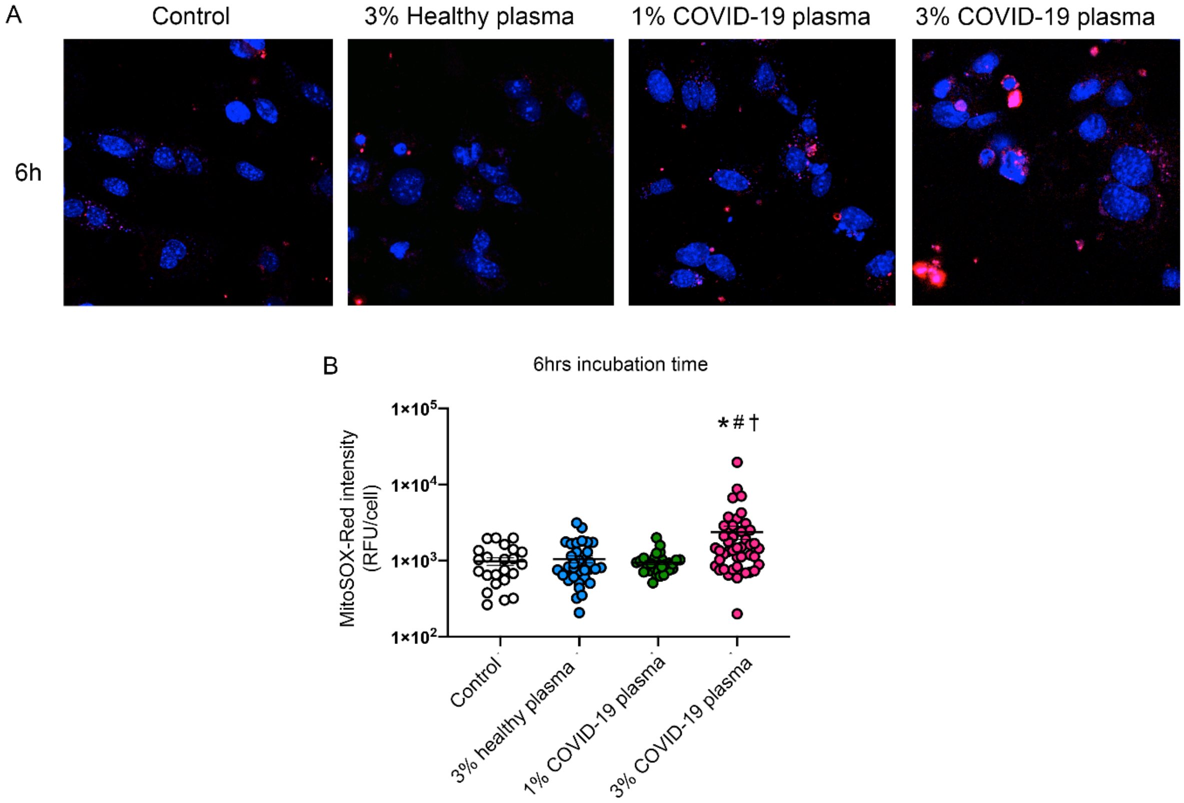
A
Control
3% Healthy plasma
1% COVID-19 plasma
3% COVID-19 plasma
6h
B
6hrs incubation time
MitoSOX-Red intensity (RFU/cell)
1×10^5
1×10^4
1×10^3
1×10^2
*#†
Control
3% healthy plasma
1% COVID-19 plasma
3% COVID-19 plasma

Supplementary Figure 4. Mitochondrial reactive oxygen species (mtROS) in mouse primary proximal tubular cells (mPPTCs) treated with COVID-19 patient B plasma of day 1.

(A) Representative images of mitochondrial superoxide, as detected by MitoSOX-Red staining and visualized under confocal microscopy. mPPTCs were untreated (control) or treated with 3% healthy volunteer plasma, 1% or 3% COVID-19 patient plasma after 6 hours of incubation. Red: MitoSOX-Red representing mitochondrial superoxide, blue: Hoechst3342 representing nuclei. Original magnification: x400. (B) MitoSOX-Red intensity in mPPTCs (n=23-44 cells from 3-5 fields/group) of each group after 6 hours of incubation. The bar in plots are expressed as mean ± standard error of the mean (SEM). Statistical significance was determined using Tukey's multiple comparison after two-way ANOVA. P-values less than 0.05 were considered statistically significant; *p<0.05: vs control, #p<0.05: vs 3% healthy plasma, †p<0.05: vs 1% COVID-19 plasma.

Supplementary Table 1. Demographic and clinical data's of Flu and RSV patients.

	Influenza (n=16)	RSV (n=9)	RSV and Influenza combined (n=21)
Sex			
Male (%)	9 (43.8)	2 (22.2)	11 (52.4)
Female (%)	7 (56.3)	7 (77.8)	10 (47.6)
Median Age (years)(IQR)	55 (25.75-62)	53 (31-61.5)	56 (31-62)
Race			
White	14 (87.5)	7 (77.8)	18 (85.7)
Black	2 (12.5)	2 (22.2)	3 (14.3)
Mean BMI (SE)	26.9 (1.4)	27.5 (0.8)	27.0 (0.95)
Obesity (%)	1 (7.7)	0 (0)	1 (5.9)
Hypertension (%)	6 (42.9)	3 (37.5)	8 (44.4)
Diabetes (%)	7 (50)	4 (50)	9 (50)
Coronary Artery Disease	2 (16.7)	1 (12.5)	3 (18.8)
Heart Failure	1 (9.1)	1 (12.5)	2 (13.3)

Supplementary Table 2. Multivariable Analysis of Nuclear cell-free DNA and Outcome (deceased vs recovered)

Variable	Odds ratio	95% Confidence Interval	p-value
ncfDNA	13.3	2.2-82.1	0.0053
Sex (Male vs Female)	1.1	0.99-1.1	0.0731
Age	1.2	0.2-6.4	0.8086
Obesity (Yes vs No)	1.6	0.3-8.4	0.6068
Cancer (Yes vs No)	0.8	0.1-12.2	0.8692

ncfDNA: nuclear cell-free DNA

Supplementary Table 3. Adjusted t-test for multiple comparison using Hommel procedure.

Figure Panel	Outcome (Hospitalized Recovered vs. Deceased		Trajectories					
	t-test, Unadjusted	t-test, Adjusted	t-test, Unadjusted			t-test, Adjusted		
			HC vs. Mild	Mild vs. Moderate	Moderate vs. Severe	HC vs. Mild	Mild vs. Moderate	Moderate vs. Severe
ncfDNA	1.08E-05	0	0.492024215	3.92E-08	3.08E-09	0.492	0	0
mtcfDNA	0.529143322	0.5291	2.17E-06	2.91E-12	0.589810172	0	0	0.5898
Monocytes	0.034047696	0.034	0.001117393	1.46E-08	0.000242643	0.0011	0	5.00E-04
Neutrophils	0.001859509	0.0019	0.002194376	3.89E-06	1.08E-10	0.0022	0	0
Erythroblast	0.018271506	0.0183	0.001281169	0.000216636	9.89E-06	0.0013	4.00E-04	0
Lymphocyte	0.790886224	0.7909	0.501921301	4.55E-06	0.830440885	0.8304	0	0.8304
Lung	0.008952128	0.009	0.016324583	1.76E-08	2.49E-07	0.0163	0	0
Heart	0.026058338	0.0261	0.052275097	0.023461693	0.005773749	0.0523	0.0469	0.0173
Hepatocytes	0.046924027	0.0469	0.025306252	4.04E-05	0.012074128	0.0253	1.00E-04	0.0241
Kidney	0.121531541	0.1215	0.470366462	1.50E-06	0.006715796	0.4704	0	0.0134
Adipocytes	0.008586377	0.0086	0.081170915	1.11E-05	0.000295646	0.0812	0	6.00E-04
Pancreas	0.014490903	0.0145	0.06668904	0.031891273	0.000765462	0.0633	0.0319	0.0023
Vascular endothelium	0.001881479	0.0019	0.051222829	5.73E-07	0.001610231	0.0512	0	0.0032
Bladder	0.015742999	0.0157	0.987042441	9.96E-09	0.002071943	0.987	0	0.0041
GIT	0.056808039	0.0568	0.054544692	0.001345637	6.97E-09	0.0545	0.0027	0
Head & Neck, larynx	0.201998734	0.202	0.763553593	9.15E-06	0.005058013	0.7636	0	0.0101
